# A New Approach to Improving Healthcare Personnel Influenza Immunization Programs: A Randomized Controlled Trial

**DOI:** 10.1371/journal.pone.0118368

**Published:** 2015-03-17

**Authors:** Larry W. Chambers, Lois Crowe, Po-Po Lam, Donna MacDougall, Shelly McNeil, Virginia Roth, Kathryn Suh, Catherine Dalzell, Donna Baker, Hilary Ramsay, Sarah DeCoutere, Heather L. Hall, Anne E. McCarthy

**Affiliations:** 1 Bruyère Research Institute, Ottawa, Ontario, Canada; 2 Department of Epidemiology and Community Medicine, University of Ottawa, Ottawa, Ontario, Canada; 3 Department of Clinical and Biostatistics, McMaster University, Hamilton, Ontario, Canada; 4 Faculty of Health, York University, Toronto, Ontario, Canada; 5 Institute for Clinical and Evaluative Sciences, Toronto, Ontario, Canada; 6 Ottawa Hospital Research Institute, Ottawa, Ontario, Canada; 7 Dalla Lana School of Public Health, University of Toronto, Toronto, Ontario, Canada; 8 St. Francis Xavier University, Antigonish, Nova Scotia, Canada; 9 Canadian Center for Vaccinology, Halifax, Nova Scotia, Canada; 10 Dalhousie University, Halifax, Nova Scotia, Canada; 11 The Ottawa Hospital, Ottawa, Ontario, Canada; 12 Bruyère Continuing Care, Ottawa, Ontario, Canada; Fondazione IRCCS Ca' Granda Ospedale Maggiore Policlinico, Università degli Studi di Milano, ITALY

## Abstract

**Background:**

Healthcare personnel influenza immunization rates remain sub-optimal. Following multiple studies and expert consultations, the “Successful Influenza Immunization Programs for Healthcare Personnel: A Guide for Program Planners” was produced. This trial assessed the impact of the Guide with facilitation in improving healthcare personnel influenza immunization rates in Canadian healthcare organizations.

**Methods:**

A sample of 26 healthcare organizations across six Canadian provinces (ON, MB, NS, BC, SK, NL) was randomized to Intervention (n=13) or Control groups (n=13). Baseline influenza immunization rates were obtained for 2008–2009; the study groups were followed over two subsequent influenza seasons. The Intervention group received the Guide, facilitation support through workshops for managers and ongoing support. The Control groups conducted programs as usual. The Groups were compared using their reported influenza healthcare personnel influenza immunization rates and scores from a program assessment questionnaire.

**Findings:**

Twenty-six organizations agreed to participate. 35% (9/26) of sites were acute care hospitals, 19% (5/26) continuing care, long-term care organizations or nursing homes, and 46% (12/26) were mixed acute care hospitals and long-term care or regional health authorities. The median rate of influenza immunization among healthcare personnel for the Intervention group was 43%, 44%, and 51% at three points in time respectively, and in the Control group: 62%, 57%, and 55% respectively. No significant differences were observed between the groups at the three points in time. However, there was a 7% increase in the median rates between the Baseline Year and Year Two in the Intervention group, and a 6% decrease in the Control group over the same time period, which was statistically significant (0.071 versus -0.058, p < 0.001).

**Interpretation:**

This pragmatic randomized trial of the Guide with facilitation of its implementation improved healthcare personnel immunization rates, but these rates continued to be sub-optimal and below rates achievable in programs requiring personnel to be immunized.

**Trial Registration:**

ClinicalTrials.gov NCT01207518

## Introduction

Healthcare systems have an ethical and moral responsibility to protect patients from transmissible diseases, including influenza [1]. Patients rely on healthcare personnel to protect them while they are vulnerable. There may, however, be a disconnect between patient expectations, the behaviour and attitudes of healthcare personnel, and organizational policies and cultures that permit unimmunized personnel to continue to work while potentially transmitting influenza to their patients.

The Canadian population is 35 million and influenza deaths varied between 370 and 4,000 deaths per year from 1976 to 2007, depending on the year and study methodology used (epidemiologic, use of laboratory confirmed hospitalized cases, or modelling) to determine population estimates of disease [2–4]. During the last five influenza seasons, starting with 2009–2010, the number of hospitalizations per season varied from a low of 2,000 patients to a high of 7,000 patients [5]. In the United States, on average, more than 200,000 people are hospitalized each year for respiratory and heart conditions, illnesses associated with seasonal influenza virus infections [6]. Further, 20–30% of patients who become infected with influenza and recover, particularly in those with underlying co-morbidities, die within the year after hospitalization (all-causes); with the suspicion that influenza is a contributing factor to their demise. At discharge, 33% are more disabled and one half never recover to their baseline level of health [7,8]. The World Health Organization estimates that the global cost of influenza is between $5.9 billion and $27.7 billion per year [9]. While the rate of transmission of influenza from healthcare personnel to patients is not fully understood, rigorous studies in long-term care show that immunizing healthcare personnel against influenza each year decreases all-cause mortality among residents—by as much as 40% in some studies [10–13]. These clinically and statistically significant results are incorrectly discounted by Thomas et al. in their recent review [14], in which they argue that the effectiveness of the vaccine should be based on deaths directly associated influenza and not total mortality. Also, importantly, the biologic rationale for healthcare worker immunization does not vary by healthcare setting. Modelling and observational studies suggest that increases in healthcare personnel immunization from any baseline will lead to incremental reduction in transmission and better patient protection; that is, optimal patient protection requires that all healthcare workers be vaccinated [15–17].

Healthcare organizations devote minimal resources to preventing influenza and to reducing or controlling healthcare-associated infection through healthcare personnel influenza immunization [18]. Across Canada, reported healthcare personnel immunization rates average between 40% and 60%, despite the influenza immunization being free to healthcare personnel and despite the efforts of healthcare organizations and public health to optimize vaccine coverage. This is in contrast to the rates of 90–100% achievable in organizations requiring healthcare personnel to be immunized annually as a condition of service [19–23].

The results of a systematic review examined voluntary healthcare personnel influenza immunization programs [24] show that in long-term care organizations, programs with more components (including education or promotion, better access to vaccines, legislation or regulation, and/or role models) had higher risk ratios favouring the Intervention group. Within hospital settings, a variety of approaches are used with inconsistent results.

The Canadian Healthcare Influenza Immunization Network conducted studies to assess the impact of the of the Ottawa Influenza Decision Aid (OIDA), a decision aid designed to assist healthcare personnel in making an informed decision about immunization, to ascertain whether its use would increase the level of confidence in healthcare personnel’s influenza immunization decision and positively affect their intent to be immunized. Among the personnel that did use the OIDA, too few moved from being uncertain to deciding to be immunized. Use of the OIDA also required significant organizational support to ensure all staff had an opportunity to take it [25].

Using the results of this previous work and in response to the need for a more organized approach by healthcare organizations, the “Successful Healthcare Personnel Influenza Immunization Programs: A Guide for Program Managers” (the Guide) and companion Tool Kit were produced (see S1 File & S2 File). These tools were based on a combination of proof-of-concept and pilot studies, systematic review findings and advice of infectious disease experts in the Canadian Healthcare Influenza Immunization Network. Proof-of-concept and pilot work using draft versions of the Guide in multiple healthcare organizations not involved in the trial described here, revealed further evidence of a paucity of effective leadership support, inadequate program resources, and absence of a systematic approach to program planning, implementation and evaluation. The Guide presents healthcare organizations with a systematic approach to planning, implementing and evaluating their campaign. The Tool Kit is designed to supplement the Guide with templates and documents that can be downloaded and customized for each site.

This trial compares the impact of using the Guide and Tool Kit with facilitation between healthcare organizations using these tools against control organizations that carried out their standard practice. The primary outcome measure was the reported healthcare personnel influenza immunization rates in 2008–2009 and 2011–2012. Secondary outcomes include assessing the organization’s ability to plan, implement, monitor, and evaluate its healthcare personnel immunization program in healthcare organizations using the Guide and Tool Kit with facilitation, as compared to organizations that carried out their standard practice.

## Methods

This multicentre, randomized controlled trial was conducted during the 2010–2011 and 2011–2012 influenza seasons. The protocol for this trial and supporting CONSORT checklist are available as supporting information; see S1 CONSORT Checklist and S1 Protocol.)

### Trial Participants

In this Trial, eligible organizations included: acute care hospitals, continuing care organizations (e.g, assisted living facilities, personal care homes, nursing homes, and long-term care organizations) and regional health authorities.

In order to recruit organizations to the trial, different recruitment processes were used. Organizations were identified through consultation with: i) the Canadian National Healthcare Influenza Immunization Network members; ii) provincial/regional governments; iii) regional infection control networks; iv) postings in bulletins, e-mail lists, web site advertisements; and v) calls to organizations identified on provincial Web sites and lists of hospitals accredited by Accreditation Canada [18]. During the recruitment period, 72 organizations expressed interest in the study.

Eligible healthcare organizations that were interested in participating in the trial were required to confirm that they: 1) regularly conducted seasonal healthcare personnel influenza immunization programs; 2) used a systematic approach to measuring immunization rates; 3) could provide immunization rates for the Baseline Year plus two intervention years; 4) agreed to be randomized to receive the Guide or no intervention (the Control organizations were promised the Guide when the trial ended); 5) would complete all questionnaires during the trial; and 6) if randomized to received the Guide, would commit to adhering to the steps in the Guide: to plan, implement, monitor, and evaluate their program.

### Intervention Group

The Guide outlines five steps to planning, implementing and evaluating a seasonal influenza immunization program for healthcare personnel. Tools and checklists are provided as supplements to the Guide (the Tool Kit), as additional resources for program managers. The Guide is available in English and French at the website www.chiin.ca.

It was assumed that successful implementation of the Guide would require:

- an organizational context that was receptive to using the Guide to improve the organization’s voluntary influenza immunization program, and
- facilitation support from outside the organization on Guide implementation and on the day-to-day operation of the program [26].

Two facilitated training workshops were held in Ottawa, Ontario and in Halifax, Nova Scotia in September 2010. Each organization was offered two workshop “seats” and the trial reimbursed the program managers for their travel expenses to attend the workshop. The full-day interactive workshops provided in-depth assistance on how to implement the steps of the Guide and use the Tool Kit. The workshops were run by members of the research team. Two organizations did not send managers to these workshops, as they joined the trial too late. However, they received phone assistance about implementing the Guide from the research team. In 2011, individual site workshops were held at each of the 13 Intervention organizations. These workshops were held to conduct on-site interprofessional team training and facilitate problem-solving specific to each site, all involving senior leaders within the organization. Throughout the two intervention years (2010–2011 and 2011–2012), the research manager, in collaboration with experts in the Canadian Healthcare Influenza Immunization Network (CHIIN), maintained an ongoing relationship with the program managers in the Intervention group and responded to questions related to influenza and influenza immunization, the Guide, or other related topics. The Intervention group program managers were encouraged to use the network to share knowledge and resources, including use of a secure Internet forum. They were instructed not to share the Guide with other organizations.

### Control Group

The organizations in the Control group implemented their campaigns as usual, without the Guide or any facilitation support.

### Data Collection

Program managers provided their organization rates for 2008–2009 (Baseline Year) and 2010–2011 and 2011–2012 (Trial years). 2009–2010 was not used in this study as a Baseline Year because of the occurance of the H1N1 pandemic which led to an atypical influenza season and,therefore, was not reflective of a standard practice season.

Each organization was responsible for calculating its rates. The trial’s primary outcome was a difference in median healthcare personnel immunization rates between the Intervention and Control groups after Year Two (2011–2012) of the trial. Also, it was hypothesized that an improvement would occur in the Intervention group between the Baseline Year and Year Two.

A program assessment questionnaire, to be completed by program managers, was sent out to all participating organizations via email in May 2012. The questionnaire assessed each organization’s ability to plan, implement, monitor, and evaluate its program, based on the “Quick Reference Checklist for Success” provided in the Guide (S1 Appendix). The checklist outlines five steps for a successful program: Step One: Identify and engage your Program team (five components); Step Two: Outline your implementation plan (six components); Step Three: Determine appropriate components and relevant tools (12 components); Step Four: Secure resources, implement and monitor (five components); and Step Five: Evaluate and celebrate (two components) (see questions in S1 Appendix). Each “yes” response to one of the 30 components of the Quick Reference Checklist for Success was scored as a 1; a “no” response was scored as a 0 (S1 Appendix). Non-responders to the initial mail-out were contacted by research staff via telephone.

### Sample Size

The sample size was calculated using an estimate that each organization would contribute at least 150 healthcare personnel to the trial each year. A sample size of 46 organizations was calculated based on the equations of Kelsey et al. for randomized clinical trial studies [27] and multiplied by the design effect to estimate the number of subjects required. It emerged that complete healthcare personnel influenza immunization information became available for each organization, so the planned cluster analysis and sample size calculation was not needed or appropriate. The measured outcome was the immunization rate of the organization. The sample size calculation was formulated to detect a 20% difference between Intervention and Control groups. The 20% difference was based on consultations with program managers, who felt that this difference would be required to justify changing practices to implement all components of the Guide in their organization.

### Interim Analysis and Stopping Rules

No deleterious effects of the intervention, such as a reduction of influenza immunization among healthcare personnel, were anticipated as a result of participation in the trial. As such, no interim analyses or stopping rules were used.

### Randomization

As the Guide was designed to be used with organizations, individual organizations were randomized to receive or not receive the Guide along with facilitation.

#### Sequence generation

Given the short recruitment period with uncertainty about the willingness of organizations to participate in the trial, as organizations indicated their willingness to participate, they were allocated to the intervention or control group using block randomization. A random number spreadsheet in blocks of two was generated in Microsoft Excel.

#### Implementation and allocation concealment

A research associate generated the allocation sequence, which was placed in sealed envelopes in the order generated in Excel. As organizations indicated their participation, they were randomized based on the date and time stamp of the completed registration form.

#### Blinding (Masking)

Organizations could not be blinded to the Intervention. Only the Intervention organizations could access the Guide on the Internet. Only the research manager, research assistants and investigators who facilitated use of the Guide within Intervention organizations were aware which organizations were Intervention organizations. The research manager responsible for randomizing each organization was blinded to the randomization algorithm.

### Statistical Methods

Characteristics of the Intervention and Control groups were obtained in the Baseline Year and analyzed using descriptive statistics. The outcome measure was immunization rate which was calculated as the proportion of healthcare personnel reporting immunization in each organization participating in the trial. Differences in immunization rates were compared between Intervention and Control groups using a Mann Whitney U test. The Mann Whitney U test does not require the assumption that immunization rates be normally distributed across organizations. Exploratory analysis indicated that the rates were not normally distributed.

A t-test was used to compare the Intervention and Control groups regarding total number of recommended Guide components (S1 Appendix) implemented during the trial. For each step, the number of components implemented (and not implemented) in the Intervention and Control groups was compared using the Fisher’s exact test.

For each of the five Guide components, the number of implemented recommendations was compared between the Intervention and Control groups using Generalized EE on binomial count data. The small number of items in each Guide component prevented the use of the t-test.

### Ethics

The Bruyère Continuing Care Research Ethics Board (Ottawa), The Ottawa Hospital Research Ethics Board (Ottawa) and Capital Health Research Ethics Board (Halifax) approved the trial protocol. Each organization was given the REB approval from these boards and support was offered to process the trial protocol through their own ethics review board. Only one site elected to do so. Given that the release of this Ethics Board site would reveal the name of the participating organization, we are not able to provide the full name of this site, but can confirm that this site received full ethics approval for their participation in the trial.

Organizations that expressed interest in the trial had to complete and sign a trial registration form outlining the terms of participation and an agreement that the organization, not just the immunization program manager, agreed to trial participation.

The unit of randomization in this pragmatic trial was the 26 health care organizations. The only data collected for the trial were aggregate data that these organizations provided. This includes the total number of employees and the number of employees that received influenza immunization. No individual employees were approached to provide any information as part of this trial. With regards to the intervention including each of the thirteen randomly selected organizations using the Guide to operate their influenza immunization program, this was a corporate decision to use the Guide. As with all other decisions regarding influenza immunization program decision-making in these organizations, requesting “consent” from individual employees is not part of the corporate decision making process. The ethics boards that approved the trial as described in the trial protocol did not request that informed consent be obtained from each of the thousands of staff employed in the 26 healthcare organizations as part of the procedures required to conduct the trial.

## Results

Of the organizations sent invitations, 72 expressed interest in the trial. Of the 72, two did not meet one or more of the inclusion criteria, seven declined to participate after initially expressing interest and 37 did not complete the trial registration form (S1 Fig.). Recruitment of organizations occurred between August and November 2010. Of the 26 organizations agreeing to participate in the trial, 13 were randomly allocated to the Intervention group and 13 to the Control group.

The 26 organizations in the trial provided immunization rates for: 2008–2009, the Baseline Year; 2010–2011, Year One; and 2011–2012, Year Two.

None of the 26 organizations were lost to follow-up, all provided immunization rates for 2008–2009, 2010–2011 and 2011–2012, and all completed the program assessment questionnaire (S1 Fig.).

More “mixed” organizations comprising regional health authorities, district health units, as well as acute care hospitals and long-term care organizations, were allocated to the Control group (54%) as compared to the Intervention group (23%) (Table 1). Most organizations were in one province (Ontario): 46% of the Control group and 62% of the Intervention group. The median number of personnel in 2011–2012 was 2,577 (124–9,260) in the Intervention group and 1,860 (190–26,992) in the Control group.

**Table 1:** Characteristics of Intervention (n = 13) and Control (n = 13) Groups by Type of Organization, Province, Number of Personnel and Baseline Immunization Rates.

Characteristic	Intervention Group	Control Group
**Type of Organization [% (n)]**		
Acute Care Hospital*	62 (8)	15 (2)
Mixed†	23 (3)	54 (7)
Continuing Care‡	15 (2)	31 (4)
**Province [% (n)]**		
British Columbia	8 (1)	15 (2)
Manitoba	8 (1)	8 (1)
Newfoundland and Labrador	8 (1)	8 (1)
Nova Scotia	8 (1)	15 (2)
Ontario	62 (8)	46 (6)
Saskatchewan	8 (1)	8 (1)
**Number of Personnel 2011–2012**		
Mean Number of Personnel Reported in 2011–2012 (personnel / number of organizations)	2,971(SD 2779)	5,950 (SD 9497)
Median	2,577	1,860
Minimum	125	190
Maximum	9,260	26,922
**Baseline 2008–2009 Healthcare Personnel Influenza Immunization Rates (%)**		
Mean Rate	49 (SD 14)	58 (SD 18)
Median	43	62
Minimum	27	29
Maximum	69	92

* includes academic teaching, pediatric and community hospitals

† includes regional health authorities and district health units

‡ includes nursing homes and long-term care facilities

Table 2 shows the immunization rates of all 26 organizations between 2008–2009 (Baseline Year) and 2011–2012 (Year Two). One of the control organizations had a decrease in rates althrough all organizations reported healthcare personnel influenza immunization rates below 90%. In each year of the trial, there was a high level of variability of immunization rates in both groups. All but two of the intervention organizations had an increase in rates through time.

**Table 2:** Influenza Immunization Rates for All 26 Organizations for 2008–2009 (Baseline), 2010–2011 (Year One), and 2011–2012 (Year Two).

Org. Code	Type*	Province	Trial Group	2008–09 Numer-ator	2008–09 Denom-inator	2008–09 Rate (%)	2010–11 Numer-ator	2010–11 Denom-inator	2010–11 Rate (%)	2011–12 Numer-ator	2011–12 Denom-inator	2011–12 Rate (%)
1	ACH	ON	Intervention	2295	4500	51%	1980	4500	44%	2205	4500	49%
2	Mixed	MB	Control	888	2899	31%	891	2899	31%	686	2899	24%
3	ACH	ON	Intervention	1399	3992	35%	1315	3992	33%	1360	4146	33%
4	Mixed	MB	Control	539	1860	29%	528	1860	28%	558	1860	30%
5	ACH	ON	Intervention	207	298	69%	147	216	68%	200	265	75%
6	Mixed	BC	Control	12074	25654	47%	11435	27730	41%	12349	26922	46%
7	ACH	ON	Intervention	3042	7187	42%	2489	6249	40%	4005	7109	56%
8	ACH	ON	Control	1765	2847	62%	1623	2847	57%	1566	2847	55%
9	Mixed	SK	Control	4247	9532	45%	3957	9564	41%	4209	9792	43%
10	ACH	ON	Intervention	2540	9575	27%	4051	9500	43%	4762	9260	51%
11	Mixed	NL	Control	1679	2883	58%	1679	2833	58%	1660	3167	52%
12	Mixed	SK	Intervention	1227	2875	43%	1089	2847	38%	1110	2577	43%
13	Mixed	MB	Intervention	322	490	66%	452	753	60%	577	788	73%
14	Mixed	NS	Control	886	1422	62%	998	1568	64%	943	1543	61%
15	ACH	BC	Intervention	510	1041	49%	460	1050	44%	573	1061	54%
16	CC	ON	Control	214	285	75%	192	285	67%	203	285	71%
17	CC	NS	Intervention	81	125	65%	89	125	71%	109	125	87%
18	CC	NS	Control	100	109	92%	92	132	70%	170	212	80%
19	ACH	ON	Control	360	535	67%	257	520	49%	297	531	56%
20	CC	ON	Intervention	86	132	65%	94	132	71%	115	132	87%
21	Mixed	NL	Intervention	1157	2878	40%	1567	3162	50%	1579	3137	50%
22	CC	ON	Control	130	176	74%	118	197	60%	127	190	67%
23	CC	ON	Control	626	869	72%	547	869	63%	582	869	67%
24	ACH	ON	Intervention	909	2435	37%	983	2405	41%	927	2517	37%
25	ACH	ON	Intervention	1287	3001	43%	1200	3001	40%	1500	3001	50%
26	Mixed	BC	Control	11955	26820	45%	10162	26453	38%	9524	26238	36%

*ACH = acute care hospitals

Mixed = acute care hospitals and long-term care or regional health authorities

CC = continuing care, long-term care, nursing homes

In Year One (2010–2011), the median rate in the Intervention group was 44% (33%- 71%) (see Table 3). This difference was not statistically significant (p = 0.90) from the median of 57% in the Control group (28%- 70%). In Year Two, the median rate in the Intervention group was 51% (33%- 71%). This difference was not statistically significant (p = 0.66) from the median of 55% in the Control group (24%- 80%). Improvement in immunization rates between the Baseline Year and Year Two (2011–2012) in the Intervention group showed an overall increase of 7.1% in contrast to a decrease of 5.8% in the Control group (p<0.001) (S2 Fig.).

**Table 3:** Difference in Median Healthcare Personnel Influenza Immunization Rates from Baseline (2008–2009) to Year Two (2011–2012).

Year	Intervention	Control	
	Median rate (range)	Median rate (range)	p-value*
2008–09 (Baseline)	43 (27 to 70)	62 (29 to 92)	0.13
2010–11 (Year One)	44 (33 to 71)	57 (28 to 70)	0.09
2011–12 (Year Two)	51 (33 to 87)	55 (24 to 80)	0.66
Rate Change from Baseline to Year Two	7.1 (-2 to 24)	-5.8 (-11 to 1·0)	0.0001

*Mann Whitney U Test

Intervention organizations implemented more components of the program assessment questionnaire than did the Control organizations (Table 4). Overall, the Intervention organizations had a significantly higher total score (25.8 versus 21.0 out of a possible score of 30; p<0.001).

**Table 4:** Comparison of the Mean Summary Scores from the Intervention and Control Group Responses to the 2012 Program Assessment Questionnaire.

Question (for details, see S1 Appendix)	Maximum Possible Score	Intervention Group Mean (SD)	Control Group Mean (SD)	p-value*
**Step One**	5	4.7	3.8	0.01
Identify and engage your program team		(0.9)	(1.5)	
**Step Two**	6	4.8	3.3	0.00
Outline your implementation plan		(1.2)	(1.1)	
**Step Three**	12	10.5	9.2	0.03
Determine appropriate components and relevant tools		(0.1)	(1.5)	
**Step Four**	4	3.4	3.0	0.33
Secure resources, implement and monitor		(1.0)	(1.0)	
**Step Five**	3	2.5	1.6	0.01
Evaluate and celebrate		(0.8)	(0.1)	
**Total**	30	25.8	21.0	0.001

*Fisher's exact test

The Intervention group implemented significantly more components than the Control group in each of Steps One, Two, Three and Five (Table 4). Only Step Four (secure, resources, implement and monitor) showed no significant difference between the two groups (p< 0.33)

A Kruskal-Wallis test indicated no significant difference in organization sizes between the Intervention and Control arms (p-value = 0.779), suggesting that institution size was not a confounding variable in this case.

## Discussion/Interpretation

The Guide was developed to support the improvement of voluntary programs in healthcare organizations at an organizational level. However, while it does provide a systematic, evidence-based approach to program improvement, even with facilitation, use of the Guide by organizations was not able to improve immunization uptake to the level that is now shown in programs in which influenza immunization is a condition of service. In the United States, policies and laws requiring influenza immunization as a condition of service for healthcare personnel have been implemented, resulting in immunization rates of greater than 90%. [19–23]. Recently, a court mediator reviewed the available evidence and ruled that British Columbia’s influenza immunization condition of service policy is fair and justified, given the volume of evidence available on the beneficial effects of the influenza immunization and the need to increase healthcare personnel rates of immunization [28].

While the Guide, Tool Kit and facilitation costs were provided to the Intervention Group, these organizations reported incurring other costs from their previous practices for planning, implementation and evaluating their program, which could be expected when transitioning to a program with the whole organization fully engaged. In reports from organizations requiring personnel to be immunized against influenza (condition of service), fewer resources were required than in traditional voluntary immunization programs [19–23,29].

Compared to the findings of our systematic review [24] and the recommendations of infectious disease experts across Canada, the trial organizations were found to not have comprehensive programs that did not focus on organizational change.

A condition of participating in the trial included the intervention organizations agreeing to keep the Guide confidential and to not share it with other organizations for the duration of the trial. They reported that at the end of the trial that they had not shared it. In addition, during the trial, no other organizations reported receiving copies of the Guide.

In this pragmatic trial, the turnover of program managers in both the Control and Intervention Groups created problems with the collection of both program implementation details and immunization rates. However, because the organization, rather than the individual manager, had committed to the trial, and, as importantly, the trial staff remained the same during the trial, organizations were able to obtain and report data required for the trial.

Originally, 2010–2011 was the time chosen to begin the intervention and 2009–2010 would have been the baseline year for comparison. As the impact of H1N1 during 2009–2010 was significant, 2008–2009 was chosen as it was more appropriate to be the baseline year. The perceptions of the dangers of H1N1 by the general community and by healthcare personnel in particular, could have resulted in different behaviours regarding immunization in 2009–2010 and beyond. Due to resource contraints, it was not possible to change the intervention start date. Therefore, it may be that 2010–2012 rates continued to be affected by H1N1. This effect was similar in both groups. The fact that organizations were randomly allocated minimized the effect of H1N1 on immunization rates.

The results of this Trial demonstrate that even with a structured approach with facilitation following the PARiHS [26] model of knowledge translation, these organizations cannot overcome the number of organizational hurdles that prevent high immunization rates. The learnings from preparatory work for this Trial demonstrated that:

1. within organization political challenges to influenza immunization of healthcare personal must be resolved including labelling activities to increase rates as a “program” and managing the program like other “programs” in the organization;
2. organizational commitment to the program from managers, leads and senior managers is required;
3. greater trust in the organization leaders who are implementing the program is required;
4. departmental silos must be removed and inter-professional/department co-operation encouraged; and,
5. there must be greater organization-wide understanding of the purpose of the immunization programs, the vaccine and its side effects.

The 46 organizations were not recruited as estimated in the trial protocol sample size calculation. Too little information from previous studies was available to guide the level of difference between groups and times to know what would be clinically/administratively significant. Also, a simple randomized trial design was chosen to be used and can lead to imbalance in the characteristics of the groups being compared. However, the sample of 26 participating organizations was sufficient to detect a statistical and a meaningful difference in change in immunization rates was observed. The small number of participating organizations precluded multi-variate analyses, as did the non-normal character of the influenza rates.

Information characterizing the 46 organizations that did not participate in the Trial was not collected. It is possible that the 26 participating organizations differ from these organizations. The size of healthcare workforce and rates of immunization of the 26 organizations were comparable to the characteristics of 721 health care organizations that participated in a recent cross Canada survey on health care personnel iinfluenza immunization [18], suggesting the results of this Trial are generalizable. Further research is required to understand the characteristics of organizations that predict use of the Guide to improve voluntary healthcare personnel influenza immunization programs.

The Intervention group in this trial reported that making the changes recommended in the Guide required substantial organizational changes. The systematic review by Lam et al. [24] found that organizations that continued to improve the content of their voluntary programs over multiple years were able to show increasing uptake of influenza immunization by their healthcare personnel; however, no organizations achieved the 90% to 100% immunization rate achieved in condition of service programs. It is possible that the 13 Intervention organizations will have greater increases in uptake of influenza immunization if they continued to use the Guide.

Assistance to facilitate implementation of the Guide requires resources from somewhere in the system. It may be that facilitation is an important intervention component, but the design of this trial did not allow for the exploration of this as a separate effect. Future studies could assess the impact of using the Guide to reduce risk of influenza outbreaks in healthcare organizations, mortality and morbidity of residents in healthcare organizations, reduction in hospitalizations and emergency department visits, and on patient flow. Also, future trials may have the resources to work with participating organizations to ensure better estimates of the numerator and denominator data used in calculating immunization rates, including rates for specific employee groups.

The Guide can be used by organizations to improve their voluntary programs and can provide tools needed to work towards building a consensus within regional or provincial jurisdictions to:

1. reach agreement on standardized methods of calculating healthcare personnel influenza immunization rates within healthcare organizations;
2. calculate influenza immunization rates consistently across organizations;
3. publicly report healthcare personnel influenza immunization rates;
4. evaluate best-practice policies;
5. evaluate the changes in these policies on an annual basis; and
6. assess trends from year to year.

A general conclusion arising from this study appears to be that immunization as a condition of service will immunize sufficient healthcare personnel each year to reduce the transmission and effect of influenza on patients and co-workers. It would be beneficial in future studies to compare voluntary and condition-of-service programs.

## Supporting Information

S1 Fig CONSORT 2010 Flow Diagram. (DOC)

S2 Fig Difference in HCP Immunization Rates (Year Two and Baseline) across Comparison Groups. (DOCX)

S1 Appendix Program Assessment Questionnaire. (DOCX)

S1 File CHIIN Guide Edition 3—English. (PDF)

S2 File CHIIN Companion Tool Kit to the Guide—English. (PDF)

S1 CONSORT Checklist (DOC)

S1 Protocol (DOC)
