# Supplementary material for: A New Approach to Improving Healthcare Personnel Influenza Immunization Programs: A Randomized Controlled Trial
Source: PLoS One. 2015 Mar 17;10(3):e0118368. doi: 10.1371/journal.pone.0118368 (PMC4363667; doi:10.1371/journal.pone.0118368)
Supplement: S1 Fig — (DOC) [file pone.0118368.s001.doc]

**S1 Figure. CONSORT 2010 Flow Diagram**

**Organizations Invited**

Organizations Expressing Interest in the Study (n=72)

**Allocation**

**Analysis**

**Follow-Up**

**Enrollment**

Excluded (n=46)

- Did not meet inclusion criteria (n=2)
- Declined to participate (n=7)
- Choose not to complete registration (n=37)

Focus of Analysis

- % of personnel immunized
- Program assessment questionnaire findings
- Lost to follow-up (n=0)
- Discontinued intervention (n=0)

Allocated to Intervention Group (n=13)

- Attended first training workshop 2010 (n=11)
- Held on-site intensive workshop 2011 (n=13)
- Completed requirements for primary outcomes analysis (n=13)
- Completed program assessment questionnaire (n=13)
- Lost to follow-up (n=0)
- Discontinued intervention (n=0)

Allocated to Control (n=13)

- Completed requirements for primary outcomes analysis (n=13)
- Completed program assessment questionnaire (n=13)

Focus of Analysis

- % of personnel immunized
- Program assessment questionnaire findings

Randomized (n=26)
