# Supplementary figures and images for: A New Approach to Improving Healthcare Personnel Influenza Immunization Programs: A Randomized Controlled Trial
Source: PLoS One. 2015 Mar 17;10(3):e0118368. doi: 10.1371/journal.pone.0118368 (PMC4363667; doi:10.1371/journal.pone.0118368)

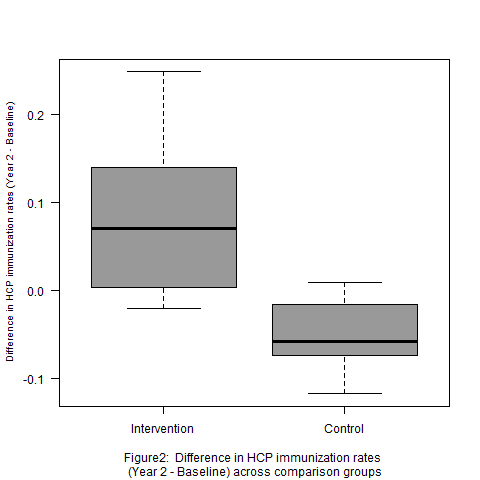

Supplement: S2 Fig — (DOCX) [file pone.0118368.s002.docx]
