# Supplementary material for: A New Approach to Improving Healthcare Personnel Influenza Immunization Programs: A Randomized Controlled Trial
Source: PLoS One. 2015 Mar 17;10(3):e0118368. doi: 10.1371/journal.pone.0118368 (PMC4363667; doi:10.1371/journal.pone.0118368)
Supplement: S1 Appendix — (DOCX) [file pone.0118368.s003.docx]

**S3 Appendix**

**Program Assessment Questionnaire: Intervention and Control Group Responses in the 2012 Survey to each Component within each of the Five Steps outlined in the Guide.**

|  | **Intervention**  **(%)** | **Control**  **(%)** |
| --- | --- | --- |
|  | **n=13** | **n=13** |
|  | **Maximum**  **Score per Question = 13** | **Maximum Score per**  **Question = 13** |
|  | **Yes = 1** | **Yes = 1** |
| **Questions** | **No = 0** | **No = 0** |
| **Step 1 - Identify and Engage your Program Team** |  |  |
| 1. Have you identified a multidisciplinary campaign team? | 13 (100) | 11 (85) |
| 1. Have you requested support from opinion leaders and senior managers? | 13 (100) | 11 (85) |
| 1. Do you have someone on your campaign team designated to coordinate the marketing and communications functions? | 12 (92) | 9 (69) |
| 1. Do you have someone on your campaign team who can assist you to determine what information you should collect? | 11 (85) | 9 (69) |
| 1. Do you have someone on your campaign team who can assist you to determine how your rates will be calculated? | 12 (92) | 10 (77) |
| **Step 2 - Outline your Implementation Plan** |  |  |
| 1. Do you have a process to obtain consent for immunization refusals (i.e. declination forms)? | 8 (62) | 5 (38) |
| 1. Have you identified potential barriers to success? | 13 (100) | 12 (92) |
| 1. Have you engaged those individuals who normally are not immunized to assist in identifying potential solutions to the barriers identified? | 8 (62) | 6 (46) |
| 1. Have you set your target immunization rate for the current campaign season (i.e. number of immunized personnel / target increase in personnel immunization over last year)? | 12 (92) | 9 (69) |
| 1. Have you incorporated the Ottawa Influenza Decision Aid as one of your tools? | 8 (62) | 1 (8) |
| 1. Have you reviewed your healthcare personnel immunization policy? | 13 (100) | 10 (77) |

| **Step 3 - Determine Appropriate Components and**  **Relevant Tools** |  |  |
| --- | --- | --- |
| 1. Do you have a policy that strongly supports influenza immunization of healthcare personnel? | 10 (77) | 11 (85) |
| 1. Does your policy include the use of declination forms? | 4 (31) | 2 (15) |
| 1. Do you have multiple clinics that are easy to access? | 13 (100) | 13 (100) |
| 1. Do your clinics offer flexible hours? | 13 (100) | 13 (100) |
| 1. Have you planned an event to launch / promote the campaign? | 11 (85) | 5 (38) |
| 1. Have you included activities that encourage immunization and involve leaders and / or senior personnel? | 12 (92) | 10 (77) |
| 1. Do you have incentives to engage the undecided? (e.g. stickers, time off, quizzes, contests, prizes, etc.)? | 10 (77) | 11 (85) |
| 1. Have you developed a broad communication plan? | 12 (92) | 9 (69) |
| 1. Is reference material accessible for personnel who may want to learn more about influenza and immunization (e.g. information on the organization’s intranet, links to websites, publications, etc.)? | 12 (92) | 13 (100) |
| 1. Do you have support and commitment from the senior medical personnel? | 13 (100) | 10 (77) |
| 1. Do you have a system in place to track and monitor the immunization results (i.e. data)? | 13 (100) | 13 (100) |
| 1. Do you have a process / formula established to calculate and compare your immunization rates? | 13 (100) | 10 (77) |
| **Step 4 – Secure Resources, Implement and Monitor** |  |  |
| 1. Have you identified the human resources you will need? | 13 (100) | 11 (85) |
| 1. Do you have enough people to get the job done? | 11 (85) | 10 (77) |
| 1. Have you identified the financial resources you will require? | 11 (85) | 9 (69) |
| 1. Have you secured the resources you need? | 9 (69) | 9 (69) |
| 1. Do you have a standardized approach to monitor healthcare personnel immunization rates? | 12 (92) | 12 (92) |
| **Step 5 - Evaluate and Celebrate** |  |  |
| 1. Have you developed an evaluation plan for the campaign? | 10 (77) | 5 (38) |
| 1. Have you planned a post-immunization campaign event / strategy to share the results with personnel? | 10(77) | 4 (31) |
