# Supplementary material for: A New Approach to Improving Healthcare Personnel Influenza Immunization Programs: A Randomized Controlled Trial
Source: PLoS One. 2015 Mar 17;10(3):e0118368. doi: 10.1371/journal.pone.0118368 (PMC4363667; doi:10.1371/journal.pone.0118368)
Supplement: S1 File — (PDF) [file pone.0118368.s004.pdf]

Canadian Healthcare
Influenza Immunization
Network

Réseau Canadien
d'Immunisation contre l'Influenza
dans les Soins de Santé

Successful Healthcare Personnel Influenza Immunization Programs

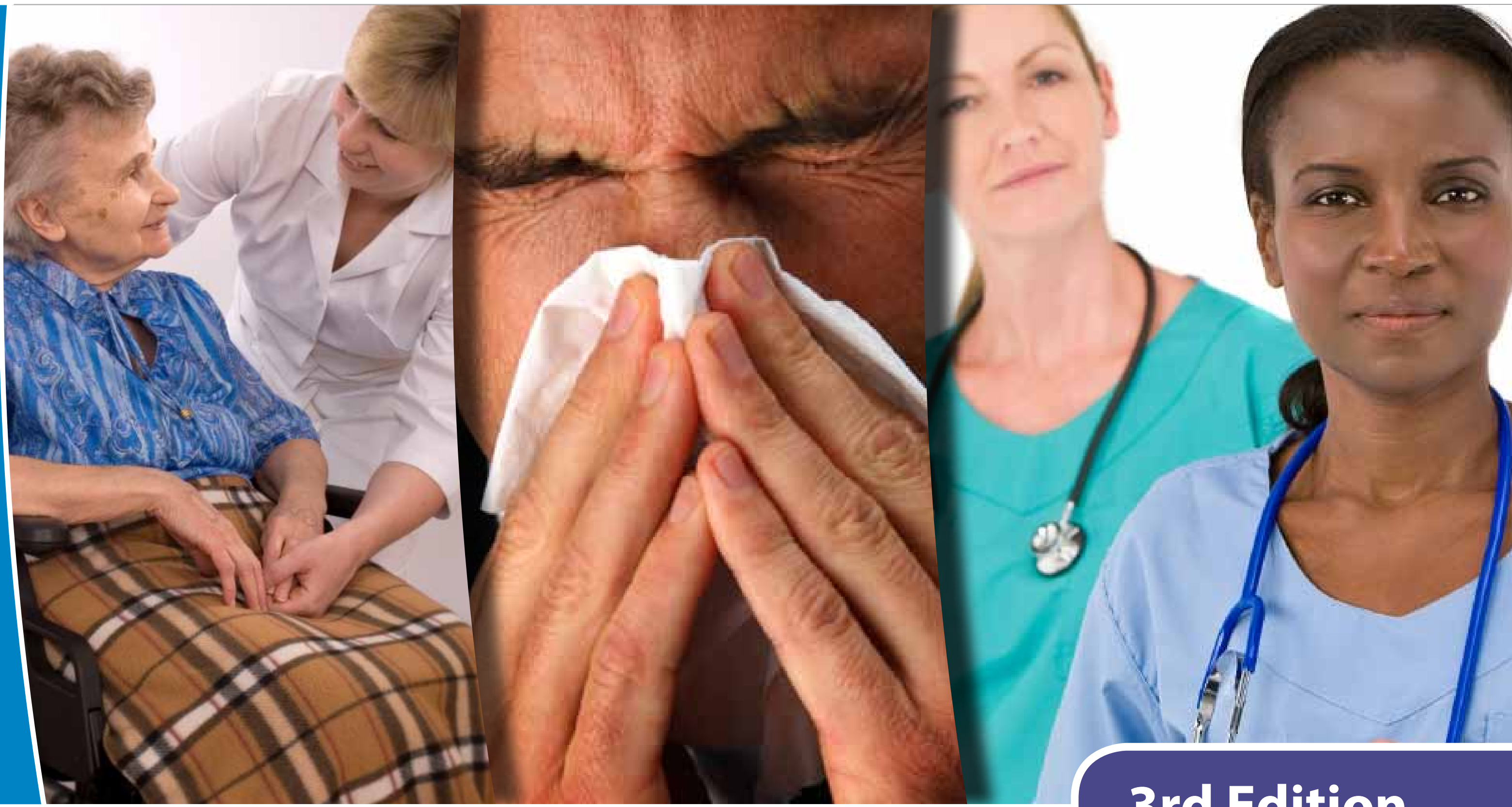

3rd Edition

A Guide for Program Planners

August 17, 2012

Edition 3 – August 17, 2012

Edition 3 of "the Guide" is the culmination of many years of research and testing in multiple settings. The Canadian Healthcare Influenza Immunization Network is primarily a network of researchers, program managers, infection prevention and control and occupational health and safety experts, policy makers, senior healthcare leaders and organizations committed to improving healthcare personnel influenza immunization rates.

This Guide was funded primarily through a grant from the Canadian Institutes of Health Research

Canadian Healthcare Influenza Immunization Network
Réseau Canadien d'Immunisation contre l'Influenza dans les Soins de Santé

CIHR IRSC
Canadian Institutes of Health Research
Instituts de recherche en santé du Canada

Patient Safety & Organizational Excellence

When developing this Guide, we conducted research studies and reviews of the scientific evidence. Our conclusion is that a robust healthcare personnel influenza immunization program:

- Is a patient quality and safety program that reduces morbidity and mortality in patients and residents and reinforces the core business of healthcare organizations
- Saves the healthcare system money
- Protects healthcare personnel as well as patients and residents
- Is increasingly seen in North America as an accreditation issue
- Is similar to existing healthcare personnel health requirements (such as requiring immunity to measles, mumps, rubella; requiring TB skin testing and requiring proper attire when attending patients and residents)
- It contributes to excellence and being a leading edge organization

Using the Guide

This Guide is meant to be used by those in healthcare organizations committed to taking a structured, evidence-based approach to improving healthcare personnel influenza immunization rates. In this third edition, we can report that the organizations who have used the Guide found it easy to use, although deceptively difficult to implement. To improve healthcare personnel influenza immunization rates, the changes needed are structural and programmatic organizational changes, as well as changes at every level of the healthcare system. A shift in organizational thinking and priority is needed. We hope that the Guide provides an easy-to-use guide as your organization navigates through these changes. We welcome feedback and ideas, and encourage everyone to check out our website www.chiin.ca for additional tools.

Tell us your stories. What worked for you, what didn't work?
What would you need that is different?

Contact us: info@chiin.ca
www.chiin.ca

Table of Contents

Acknowledgments **4**

Introduction **7**

Definitions **9**

Quick Reference Checklist for Success **11**

The 5 Steps to Planning an Effective Immunization Program **13**

Step 1 ▶ Identify and Engage Your Program Team 14

Step 2 ▶ Outline Your Implementation Plan 18

Step 3 ▶ Determine Appropriate Components and Relevant Tools 24

Step 4 ▶ Secure Resources, Implement, and Monitor 26

Step 5 ▶ Evaluate and Celebrate 28

Conclusion **30**

Appendix 1 ▶ **Summary of the Research Findings** **31**

Appendix 2 ▶ **Calculating Rates** **32**

Appendix 3 ▶ **Patient Contact Categories** **35**

Appendix 4 ▶ **The Ottawa Influenza Decision Aid** **36**

Appendix 5 ▶ **Outcome Evaluation Examples** **37**

Appendix 6 ▶ **Step-By-Step Approach to Evaluation** **39**

Appendix 7 ▶ **Planning Calendar Template** **40**

References **45**

Acknowledgments

The Canadian Healthcare Influenza Immunization Network team would like to thank the following individuals and organizations for their support in the development of this Guide.

Working Group – 2nd Edition

- Larry W. Chambers, Bruyère Research Institute
- Lois Crowe, Bruyère Research Institute
- Caroline George, Bruyère Research Institute
- Heather L. Hall, Bruyère Continuing Care
- Elina Farmanova-Haynes, Bruyère Research Institute
- Alexandra Henteleff, Innovative Solutions Health Plus
- Po-Po Lam, Bruyère Research Institute
- Helena Wall, Innovative Solutions Health Plus

Working Group – 3rd Edition

- Irene Armstrong, Toronto Public Health
- Larry W. Chambers, Bruyère Research Institute
- Lois Crowe, Bruyère Research Institute
- Po-Po Lam, University of Toronto
- Allison McGeer, Mt. Sinai Hospital
- Paula Neves, Ontario Long Term Care Association

Contributors

- Donna Baker, Bruyère Continuing Care
- Kevin Best, Ottawa Hospital Research Institute
- Sherry Bowman, St. Francis Xavier University
- Rebecca Cassidy, Ottawa Hospital Research Institute
- Sarah DeCoutere, Capital Health
- Kieran Jordan, Bruyère Continuing Care
- Chantal Lafleur, Bruyère Research Institute
- Hilary Ramsay, Bruyère Research Institute
- Jeremy Levine, Bruyère Research Institute
- Anne McCarthy, Ottawa Hospital Research Institute
- Allison McGeer, Mt. Sinai Hospital
- Shelly McNeil, Canadian Center for Vaccinology
- Sarah Kwan Mulvihill, Bruyère Research Institute
- Donna Pierrynowski-MacDougall, Canadian Center for Vaccinology
- Joanne A. Villeneuve, Bruyère Continuing Care
- Craig White, Canadian Center for Vaccinology

Contributing Organizations

The following organizations have supported this research project and have contributed significantly to the development and content of this Guide:

- Alderwood
- Annapolis Valley Health
- Barry's Bay Memorial Hospital
- Brandon Regional Health Authority
- Bruyère Continuing Care
- Bruyère Research Institute

- Burntwood Regional Health Authority
- Canadian Blood Services
- Canadian Center for Vaccinology
- Canadian Institutes of Health Research
- Carleton Place and District Memorial Hospital
- Central Healthcare
- Children's Hospital of Eastern Ontario
- Finlandia Nursing Home
- Fraser Health
- Grey Bruce Health Services
- Guysborough Antigonish Strait Health Authority
- Huron County Health Unit
- Immunize Canada
- Interlake Regional Health Authority
- IWK Health Centre
- Lee Manor
- London Health Sciences Centre
- North York General Hospital
- Northern Health
- Northumberland Hills Hospital
- Northwood Care
- Ontario Long Term Care Association
- Ontario Ministry of Health and Long-Term Care
- Ottawa Hospital Research Institute
- Ottawa Public Health
- Public Health Agency of Canada/Canadian Institutes of Health Research Influenza Research Network
- Perley and Rideau Veterans' Health Centre
- QEII Health Sciences Centre / Capital Health
- Regina Qu'Appelle Health Region
- Rideaucrest Home
- Rockwood Terrace - County of Grey
- Shannex Arborstone
- Shannex Bisset Court
- Shannex Caritas Residence
- Shannex Glasgow Hall
- Shannex Mapleston
- Shannex Parkland at the Lakes
- Shannex Parkston
- St. Boniface Hospital
- St. Joseph's General Hospital, BC
- St. Joseph's Healthcare London
- Summit Place
- Sunrise Health Region
- Sunrise Health Region
- The Hospital for Sick Children
- The Mira
- The Ottawa Heart Institute
- The Ottawa Hospital
- The Ottawa Hospital Influenza Committee
- The Ottawa Hospital, Hotel Services
- Thunder Bay Regional Health Sciences Centre
- West End Villa
- Western Health
- Winchester and District Memorial Hospital
- Woodingford Lodge Long Term Care Facility

Introduction

Why Would You Want to Use this Guide?

This innovative Guide provides program managers (including occupational health and safety, infection prevention and control, and administration managers) and program teams with a comprehensive, straightforward, and guided approach to building a successful influenza immunization program based on current evidence.

Current initiatives aimed at increasing healthcare personnel immunization rates have been limited in their success. Both the National Advisory Committee on Immunization and Accreditation Canada recommend that all healthcare organizations use up-to-date, evidence-based approaches and policies to improve seasonal influenza immunization rates (1, 2).

When the influenza vaccine is provided to healthcare personnel, frequency and severity of influenza outbreaks are reduced, as are influenza-associated illness and death among patients or residents due to decreased transmission of influenza from healthcare personnel to their vulnerable patients or residents. Healthcare personnel immunization minimizes absenteeism and reduces the financial burden on healthcare organizations (3).

Despite an abundance of evidence about the safety and effectiveness of the influenza vaccine (4-7), immunization rates among healthcare personnel in hospital and long-term care settings remain well below the public health target of 100%. Healthcare personnel immunization rates are as low as 2%, but average between 40% and 60% for most healthcare organizations (3, 4, 8).

Infectious disease & public health experts recommend that all, or 100%, or healthcare personnel be immunized.

Historically, rates average between 40% and 60% (9).

How Was this Guide Developed?

Our Canadian team of researchers developed a tool, called the Ottawa Influenza Decision Aid (OIDA) (10), to help undecided healthcare personnel make an informed choice about whether or not to be immunized. During the first year of the project, it became clear through consultations that managers responsible for influenza immunization programs in healthcare organizations devoted significant effort to increase immunization rates, with limited success.

As a result, the team conducted scientific reviews of the literature (11) to identify factors and elements that might contribute to building a successful influenza immunization program within a healthcare organization. The "Successful Healthcare Personnel Influenza Immunization Programs: A Guide for Program Planners" (the Guide) is a result of those consultations and research.

A summary of the research findings that inform this Guide can be found in Appendix 1.

What Have Others Said About the Guide?

Between 2010 and 2012, 13 healthcare organizations used the Guide to improve their healthcare personnel influenza immunization programs. Results from this two-year, cluster randomized controlled trial are currently being analyzed. Overall, program managers using the Guide said they found it helpful and easy-to-use. Preliminary results are promising, but we learned that, not surprisingly, improvement in a voluntary program takes effort, commitment from the right people and time to improve. Check out our website at www.chiin.ca in late fall 2012 for updates.

In 2009, this Guide was pilot-tested and updated based on the feedback that was received. **Of the 10 organizations that pilot-tested the Guide and provided feedback, 100% indicated they would recommend it to other organizations and 90% stated they made changes based on the information contained within the Guide.**

Comments received from users of the Guide:

> "Provides a structured and planned approach to ensure everything is covered"
>
> "Helped us to approach program in a planned way"
>
> "Provided pre-planning ideas that resulted in better communication and dialogue from employees"
>
> "I felt better prepared to research new resources and build on old ones"
>
> "Although this Guide was developed to support annual influenza immunization programs, most of the principles can be applied to other immunization programs"

How Can I Get More Information?

This Guide is accompanied by a web-based toolkit with "grab-and-go" items that managers can easily use and modify. It is anticipated that this toolkit will continue to grow as organizations add the creative tools they have developed. The latest edition of the Guide and toolkit can be found on the Canadian Healthcare Influenza Immunization Network (CHIIN) website at www.chiin.ca.

We encourage you to visit the Canadian Healthcare Immunization Influenza Network (CHIIN) website at www.chiin.ca to find out what other organizations like yours have done to improve their healthcare personnel immunization rates, to exchange what you are currently doing, and to join in the various discussions.

Definitions

Healthcare Personnel

The term healthcare personnel (HCP) is defined broadly as all paid and unpaid persons working in healthcare settings (12).

Healthcare Organization (or Setting)

Healthcare personnel work in a variety of settings, including (but not limited to) acute care hospitals, long-term care facilities, skilled nursing facilities, rehabilitation centres, urgent care centres, outpatient clinics, home and community healthcare agencies, and emergency medical services (12).

Immunization and Vaccination

The terms immunization and vaccination are often used interchangeably. For the purpose of this Guide, the term immunization will be used.

> **Immunization** (syn. Vaccination) Protection of individuals from communicable disease by administration of a living modified agent …, a suspension of killed organism…, or an inactivated toxin (13).
>
> **Vaccination** Strictly speaking, vaccination refers to inoculation with vaccinia virus against smallpox. Nowadays the word is broadly used synonymously with procedures for immunization against all infectious disease (13).

In-Kind Support

In-kind support is a cash-equivalent contribution in the form of an asset for which no cash is exchanged. In-kind resources might be things you would otherwise pay for, or they might be things that money just cannot buy. When someone offers to provide a service, supplies, or help, you are receiving in-kind support. You can look for in-kind support from within your organization and your local community. In-kind support is as equally important as financial support. Seeking in-kind support should be an integral part of your plan for action and sustainability (14).

Some healthcare personnel provide direct patient care. Other HCP, such as housekeepers, maintenance personnel, vendors, volunteers, or outside contractors, may not provide direct patient care, but have jobs that may put them into close contact with patients or residents or their environment (12).

The 5 Steps to an Effective Immunization Program

Step 1

Identify and Engage Your Program Team

- Establish your team
- Define core roles
- Define other roles
- Seek potential people to recruit
- Identify attributes to look for

Step 2

Outline Your Implementation Plan

- Review your organization's personnel influenza immunization policy
- Identify strengths, weaknesses, opportunities, and threats
- Identify barriers to implementation and immunization
- Identify program goals and objectives

Step 3

Determine Appropriate Components and Relevant Tools

- Legislation / regulation
- Improved access
- Education / promotion
- Role models
- Measurement and feedback

Step 4

Secure Resources, Implement, and Monitor

- Secure resources
- Implement your plan
- Monitor your progress

Step 5

Evaluate and Celebrate

- Reflect on and evaluate your program
- Celebrate

Quick Reference Checklist for Success

			Yes	No
**Step 1**	1.	Have you identified your multidisciplinary program team?	☐	☐
	2.	Have you requested support from opinion leaders and senior managers?	☐	☐
	3.	Do you have someone on your program team designated to coordinate the marketing and communications functions?	☐	☐
	4.	Do you have someone on your program team who can assist you to determine what information you should collect?	☐	☐
	5.	Do you have someone on your program team who can assist you to determine how your rates will be calculated?	☐	☐
**Step 2**	6.	Do you have a process to obtain consent for immunization refusals (i.e. declination forms)?	☐	☐
	7.	Have you identified potential barriers to success?	☐	☐
	8.	Have you engaged those individuals who normally are not immunized to assist in identifying potential solutions to the barriers identified?	☐	☐
	9.	Have you set your target immunization rate for the current program season (i.e. number of immunized personnel / target increase in personnel immunization over last year)?	☐	☐
	10.	Have you incorporated the Ottawa Influenza Decision Aid as one of your tools?	☐	☐
	11.	Have you reviewed your healthcare personnel immunization policy?	☐	☐
**Step 3**	12.	Do you have a policy that strongly supports influenza immunization of healthcare personnel?	☐	☐
	13.	Does your policy include the use of declination or decision forms?	☐	☐
	14.	Do you have multiple clinics that are easy to access?	☐	☐
	15.	Do your clinics offer flexible hours?	☐	☐
	16.	Have you planned an event to launch / promote the campaign?	☐	☐
	17.	Have you included activities that encourage immunization and involve leaders and / or senior personnel?	☐	☐
	18.	Do you have incentives to engage the undecided? (e.g. stickers, time off, quizzes, contests, prizes, etc.)?	☐	☐

		Yes	No
	19. Have you developed a broad communication plan?	☐	☐
	20. Is reference material accessible for personnel who may want to learn more about influenza and immunization (e.g. information on the organization's intranet, links to websites, publications, etc.)?	☐	☐
	21. Do you have support and commitment from the senior medical personnel?	☐	☐
	22. Do you have a system in place to track and monitor the immunization results (i.e. data)?	☐	☐
	23. Do you have a process / formula established to calculate and compare your immunization rates?	☐	☐
**Step 4**	24. Have you identified the human resources you will need?	☐	☐
	25. Do you have enough people to get the job done?	☐	☐
	26. Have you identified the financial resources you will require?	☐	☐
	27. Have you secured the resources you need?	☐	☐
	28. Do you have a standardized approach to monitor healthcare personnel immunization rates?	☐	☐
**Step 5**	29. Have you developed an evaluation plan for the program?	☐	☐
	30. Have you planned a post-immunization campaign event / strategy to share the results with personnel?	☐	☐

Please see Appendix 6 for a detailed planning calendar template.

The 5 Steps to Planning an Effective Immunization Program

Healthcare organizations are complex entities and any change will take time to implement. We suggest that you consider adopting one or two changes to your program in any given year.

Sequentially implementing small changes, each of which is not that costly, will help to slowly but steadily increase your success over time.

In order to improve the program's impact and value to the organization, regardless of the type of healthcare organization involved, key issues should be addressed annually before and after each campaign. Each step outlined below presents a starting point for discussion. Recognizing that there is no one right way to do things, the concept behind this Guide is to provide the best available evidence on how to run a successful program.

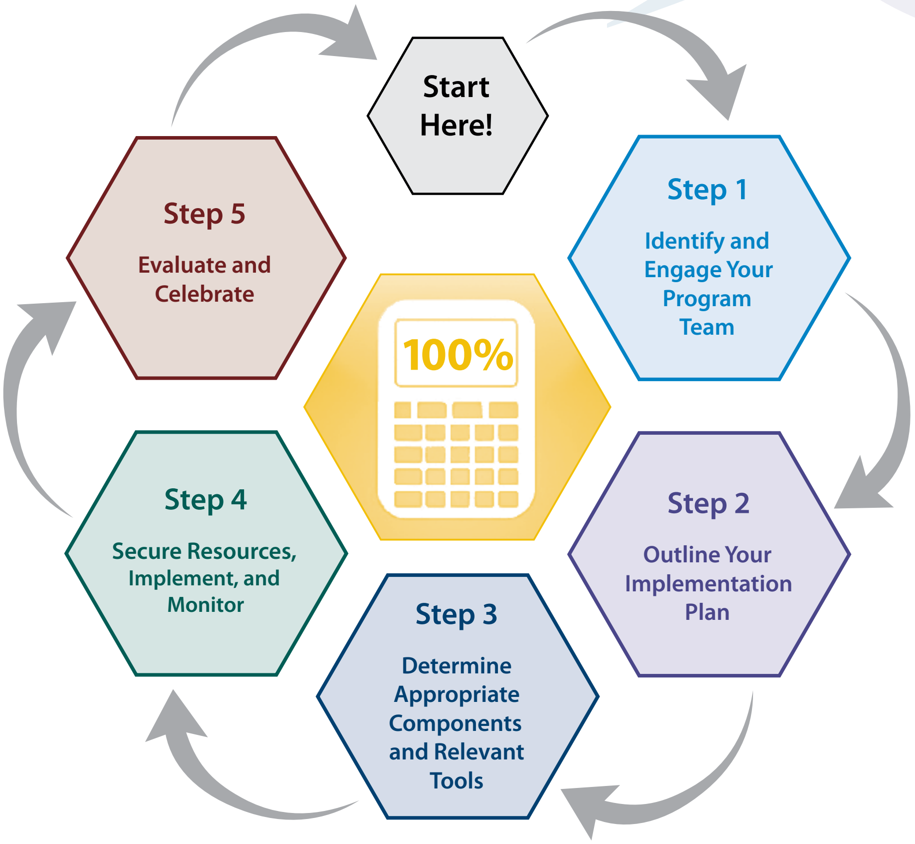

Calculating rates has been identified as one of the biggest challenges organizations encounter. Look for this symbol throughout the Guide and refer to Appendix 2 for more information about calculating rates.

Organizations will be at different stages of readiness and must determine where to focus their efforts and prioritize their goals.

Step 1 — Identify and Engage Your Program Team

- Establish your team
- Core program team roles
- Other roles
- Potential people to recruit
- Attributes to look for

Establish Your Team

Establishing a program team is the critical first step for a successful influenza immunization program. Careful consideration must be given to ensure you are strategic in inviting influential people to be part of the program team – for example, decision makers, informal leaders, opinion leaders, gatekeepers, and other key representatives.

The role of opinion leaders and physicians is very important and cannot be overlooked. It is particularly important to engage opinion leaders and physicians during the planning and implementation stages.

It is important to recognize that members of the program team will have numerous "regular" work commitments and busy work schedules. Therefore, requests for participation should be targeted and contributions acknowledged.

The roles identified below are not mutually exclusive. Depending on the size of the organization and resources available, one person may have multiple roles at any given time.

Core Program Team Roles

Title	Role Description
Team Leader	▪ Lead, advocate, and promote the program
Administrative Support	▪ Take meeting minutes<br>▪ Communicate minutes and records of decisions<br>▪ Maintain a master file of resource materials
Senior Administration Sponsor	▪ Advocate for the project at the organizational level
Nursing Team Members	▪ Support the development and review of the clinical action plan<br>▪ Recruit unit-level contacts for communication and dissemination of information<br>▪ Administer the vaccine
Communications	▪ Assist with the development and implementation of the influenza immunization program communication plan<br>▪ Format and / or acquire educational material<br>▪ Promote events such as campaign kick-off or end-of-campaign celebration
Pharmacy	▪ Assist with cold chain / vaccine delivery issues<br>▪ Assist with outbreak management planning and implementation
Information Technology Representative	▪ Assist with the development and maintenance of the database system

Epidemiologist or Statistician	▪ Assist the team to determine what information they would like to collect , monitor, report, and evaluate<br>▪ Identify what questions the team would like to answer so that the right type of information and level of detail is collected from the beginning of the program<br>▪ Assist the team to determine how the immunization rate will be calculated<br><br>See Appendix 2 for more information about calculating rates

Other Roles

Title	Role Description
Allied health and front-line support workers	▪ Support healthcare personnel<br>▪ Immunization at the frontline / unit level<br>▪ Professional team engagement (such as physiotherapy)
Union Representative / Workplace Health and Safety Committee Members	▪ Identify potential union issues and solutions early in the planning phase
Volunteers	▪ Welcome people to the clinic<br>▪ Perform administrative duties (i.e. assembling information packages and handing out consent forms during immunization sessions)<br>▪ Assist with the security and flow of the clinic

Potential People to Recruit

- Occupational Health and Safety representative
- Chief Executive Officer or Senior Vice President
- Representatives from all levels and programs across the healthcare organization
- People who are qualified to administer the vaccine
- Gatekeepers and opinion leaders
- People who know how to maintain cold chain
- Pharmacy representative
- Communications representative
- Frontline / unit level personnel

- Joint union and occupational health and safety committee member
- Information technology representative

Team Member Attributes to Look for

- Advocates influenza immunization and is committed to increasing influenza immunization rates
- Able to leverage or commit support for program, including allocation of resources and ensuring support from all levels of management
- Is a positive role model
- Has experience in communications
- Understands how to effect change
- Is well-organized and able to keep on task
- Understands the importance of resolving union related issues early in the process
- Able to transfer knowledge to information technology staff to facilitate the development of a user-friendly database that will produce useful reports
- Able to help the team prioritize activities

When inviting individuals to participate, they should be made aware that their level of involvement will ebb and flow, depending on the stage of the program. They will only need to participate during those times when their input is required.

Step 2 ▶ **Outline Your Implementation Plan**

Some items to consider when designing your implementation plan:

- ▶ **Review your organization's personnel influenza immunization policy**
- ▶ **Identify strengths, weaknesses, opportunities, and threats**
- ▶ **Identify barriers to implementation and immunization**
- ▶ **Identify goals and objectives**
- ▶ **Set your "launch date"**

It is important to do a thorough assessment to create a snapshot of your current situation to identify organizational readiness and to outline your objectives.

Review Your Organization's Personnel Influenza Immunization Policy

A comprehensive and effective organizational personnel influenza immunization policy is required to maintain a functional immunization program. Accreditation Canada has identified this as a Required Organizational Practice (ROP) (2). These policies are often affected by political, social, and economic factors, which can result in significant policy changes, and even new policy creation, from year to year.

When developing an influenza immunization policy, it is important to define which individuals should be targeted and how the immunization rate will be calculated, as well as whether volunteers should be included.

See Appendix 2 for more information about calculating rates.

Make sure policy changes are practical and that you have support from the necessary people / departments within your organization (e.g. management committee, unions, etc.).

Thorough knowledge and understanding of your current influenza immunization policy information and influences may help to identify potential strengths and weaknesses within the existing program. Knowing this information will also enable you to inform healthcare personnel about policy details and the rationale behind any changes implemented.

Consider addressing low immunization rates in novel ways, such as introducing an influenza immunization tracking form that includes consent, record of immunization elsewhere, and the option to decline (see sample in toolkit).

Identify Strengths, Weaknesses, Opportunities, and Threats

A simple and useful method for identifying existing program strategy successes, challenges, and barriers is the SWOT analysis. SWOT is a simple tool that allows program teams to analyze their situation in terms of Strengths, Weaknesses, Opportunities, and Threats.

**S**trengths	**W**eaknesses
**O**pportunities	**T**hreats

Strengths are elements that work in your favour and can be controlled.

Weaknesses are elements that require improvements and are within your control.

Opportunities are elements out of your control that could yield positive results in the future.

Threats are elements out of your control that act as barriers to possible success.

The results of this analysis may be useful to organizations when developing or restructuring their immunization programs.

For more information about how to conduct a SWOT analysis, visit:

http://www.axi.ca/tca/sep2003/facilitationrole_1.shtml
http://www.planonline.org/planning/strategic/swot.htm
http://forlearn.jrc.ec.europa.eu/guide/2_design/meth_swot-analysis.htm
http://afc.uwaterloo.ca/PDF/Building%20Blocks%20SWOT%20analysis.pdf

To obtain a blank SWOT analysis template, go to:

http://www.businessballs.com/free_SWOT_analysis_template.pdf

Identify Barriers to Implementation and Immunization

There are many barriers to implementation and immunization that have been studied in various healthcare settings. Commonly encountered barriers can be grouped according to those factors which originate within (internal) and outside (external) an organization.

Internal Barriers (Barriers You Can Control)

Individual	Organizational
▪ Mistrust of information provided<br>▪ Fear of the vaccine's side effects<br>▪ Fear / dislike of needles<br>▪ Perceived low risk-to-benefit ratio<br>▪ Poor knowledge of vaccine<br>▪ Poor knowledge of effects of influenza<br>▪ Decisional conflict (undecided)	▪ Lack of program resources<br>▪ Lack of interest from various levels of management<br>▪ Lack of understanding of program team roles

External Barriers (Barriers Outside Your Span of Control)

Individual	Organizational
▪ Misinformation from the internet<br>▪ Vaccine-influenza strain mismatch<br>▪ Inadequate vaccine supply	▪ Negative media coverage<br>▪ Confusing or contradictory media coverage<br>▪ Inadequate vaccine supply

Each organization will have a unique set of barriers and challenges to face, and these may change from year to year. Early and accurate recognition of these barriers may improve the immunization experience for program organizers and healthcare personnel alike.

To better understand the reasons why some HCP choose not to be immunized, it is important to find a way to engage them in conversations about immunization. By understanding the reasons why people choose not to be immunized, you will be able to develop strategies and approaches that will be more effective. When approaching individuals who have previously decided not to be immunized, it is important to be respectful of personal choice, privacy, and disclosure issues.

Current research indicates that decisional conflict may be a factor contributing to poor vaccine uptake and may result in individuals remaining undecided. Decision aids are tools that help with healthcare decisions and are designed to complement, rather than replace, counselling from a health practitioner.

They are designed to facilitate a better understanding of an individual's values as they relate to the risks and benefits of the options available and have been shown to resolve decisional conflict (15).

A Tool for the Undecided

Recognizing that personal values and informed choice are key factors that influence individual decisional conflict, the Ottawa Influenza Decision Aid (OIDA) was developed in 2006 by the Canadian Healthcare Influenza Immunization Network (CHIIN) team.

The Ottawa Influenza Decision Aid (OIDA):

- Recognizes personal knowledge and values
- Provides clear evidence
- Supports informed decision-making

Further information about the OIDA can be found in Appendix 4.

Identify Program Goals and Objectives

Once you have reviewed your organization's policy, completed a SWOT analysis and identified potential barriers, the next step is to clearly articulate the goals and objectives you want to achieve.

Goals are general statements of what a project is trying to do.

Objectives are specific, measurable statements of the desired change(s) that a project intends to accomplish within a given timeframe (16).

GOALS ARE THE "WHAT" OBJECTIVES ARE THE "HOW".

Evaluation is not something that happens at the end of a project. It is a process that starts with the development of goals and objectives, and continues throughout the life of the project. Through the evaluation process, we learn whether projects are meeting their goals and having an impact (16).

Objectives that are "SMART" (Specific, Measurable, Attainable, Realistic, and Time-Oriented) are more likely to be achieved.

Letter	Concept	
S	**S**pecific	Be specific about what you want to achieve
M	**M**easurable	Quantify your objective
A	**A**ttainable	Make sure what you are attempting to do is achievable
R	**R**ealistic	Make sure your objective is realistic and that you have the necessary resources
T	**T**ime-Oriented	State a timeframe to achieve the objective

Examples of Influenza Immunization Program Goals and "SMART" Objectives:

Example #1	
**Goal**	**Objective**
To increase the uptake of influenza immunization	Our program will include three components: education, testimonials from leaders, and mobile carts to increase the uptake of influenza immunization by 10% this year.

Example #2	
**Goal**	**Objectives**
To calculate an accurate influenza immunization rate for our organization	1. Our program team will establish a systematic way to count the number of paid and unpaid personnel for this year's program.<br>2. Our program team will establish a comprehensive method to determine who was and who was not immunized this year so year-to-year comparisons can be made.

Example #3	
**Goal**	**Objectives**
To communicate the influenza immunization rates at selected intervals to the appropriate informal and formal entities	1. Report immunization rates at regular intervals to staff, managers, and senior administrators during and after this year's program.<br>2. Comply with formal reporting requirements of public and government agencies.

Set Your Launch Date

An important step in planning your program is setting your launch date. Once this date is chosen, you may begin to draft a timeline with target dates for each of the tasks or milestones. It is important to sketch out your timeline to avoid tight deadlines and unrealistic expectations.

Step 3 Determine Program Components and Relevant Tools

Identified below are the five broad components for a successful influenza immunization program (11):

Component	Operational Definition
Legislation & Regulation<br>(Influence or change HCP immunization rates)	Interventions involving changes in immunization policy for healthcare personnel
Improved Access<br>(Enhance access to immunization)	Strategies to allow for easier access to immunization for healthcare personnel
Education & Promotion<br>(Organize efforts to raise awareness and / or increase knowledge of influenza immunization)	Organized effort to raise awareness and increase knowledge about influenza and influenza immunization
Role Models<br>(Involve leaders and senior personnel to encourage HCP to get immunized)	Activities that involve leaders and/or senior staff to encourage immunization
Measurement & Feedback<br>(Track immunization rates and disseminate results)	Tracking of immunization rates of healthcare personnel and dissemination of results<br>**See Appendix 2 for more information about calculating rates**

This list is not exhaustive. For more information about the program components, please see the article by Lam et al. published in the Canadian Medical Association Journal, July 2010 (11) or **see Appendix 1 for a summary of the research findings that informed this Guide**.

Before adopting any new approach, please ensure that they are aligned with your organization's policies and procedures.

Consider All Parts of the Puzzle When Looking to Improve Healthcare Personnel Immunization Rates

Examples
- Immunization advocates
- Public support from leaders
- Visible immunization of senior administrator

Examples
- Regular monitoring of immunization rates
- Reporting of coverage rates to administration and HCP
- Pre- and post-campaign worksheet to calculate immunization rate
- Post-campaign announcement

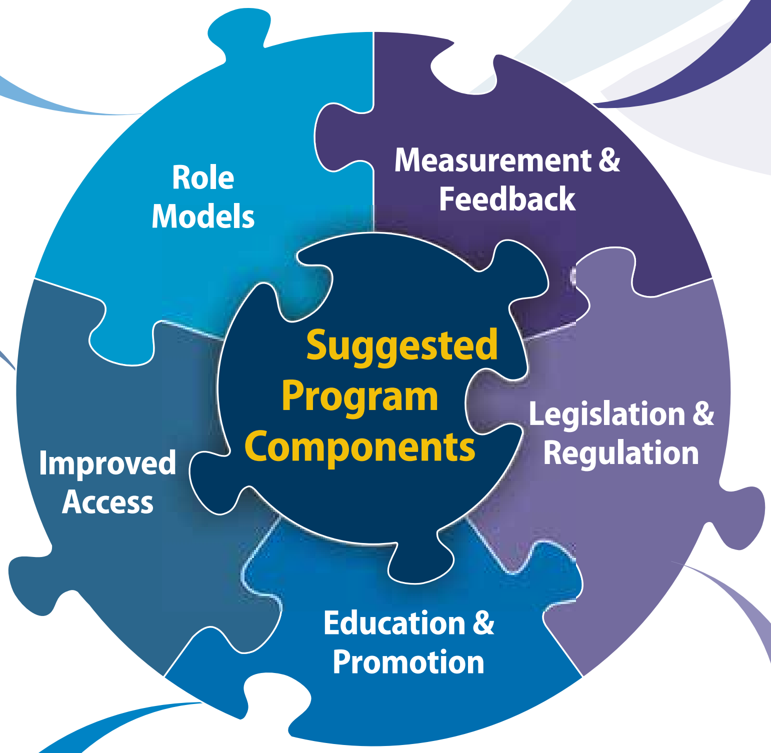

Examples
- Mobile vaccine carts
- Peer-to-peer immunization
- Additional or extended vaccine clinics

Examples
- Group educational sessions
- Incentives for receiving vaccine
- Pamphlets
- Ottawa Influenza Decision Aid

Examples
- Staff immunization policy
- Mandatory immunization policy
- Declination form

Remember to think about your audience and tailor your approaches to that group.

Step 4 | **Secure Resources, Implement, and Monitor**

Secure Resources

Every healthcare organization struggles with a lack of resources and must consider the best way to use its available resources.

> **Influenza immunization programs are often run by individuals who already have a full workload and are taking on additional work to plan and implement an influenza immunization program. This can be challenging. Consideration should be given to alternative and creative ways of maximizing resources, including partnering / consulting with other organizations, in order to maximize efficiencies and minimize duplication.**

During this phase, you will need to develop a plan that outlines what needs to be done and justifies the human and physical resources required. Consider working with your senior administration sponsor(s) to reallocate internal resources. The component or combination of components you choose will determine the resources that may be required. This includes developing and securing a budget that covers the financial resources needed and exploring alternative funding and partnership opportunities.

Examples of some of the **organizational support** that may be required:

- Nursing staff time dedicated to immunization
- Administrative support (e.g. data entry, preparation of information packages)
- Commitment and participation of CEO / Senior VP to support and promote activities for healthcare personnel influenza immunization (including asking them to take an active role in encouraging personnel to get immunized and attend special events)
- Partnering with other organizations to share resources / strategies
- Statisticians, epidemiologists, or someone who can verify that you are asking the right questions and collecting the correct data
- Departmental in-service training
- Orientation sessions for new personnel
- Development of an effective healthcare personnel influenza immunization policy
- Leadership education sessions regarding the importance of influenza immunization

Examples of **additional resources** that may be required:

- Promotional items (e.g. print resources, incentives such as badges and pins)
- Communication costs (e.g. public service announcements, promotional videos)
- Information technology costs (i.e. changes to database in order to track who has been immunized)

- Vaccine and immunization supply costs (including instruments required for immunizations, such as needles, antiseptic, etc.)
- In-kind resources and contributions
- Cost of meetings to explore partnership and funding opportunities

Significant improvements can be made with limited resources!

Adopt a can-do attitude and be creative when seeking out resources from within and outside of your organization.

Implement

Once you have gathered your program team, outlined your plan and secured the necessary resources, it is time to implement your plan. This stage is where all your planned activities and creative ideas are put into action.

Monitor

During the influenza immunization campaign, continually monitor and assess your progress by:

- Tracking immunization rates and comparing them to your targets
- Setting up daily tracking documentation processes to capture the total number of attendees, number of attendees by employment grouping, number of attendees by unit, etc.
- Setting up a system to advise personnel of daily / weekly immunization program progress
- Continually evaluating progress and the success / failure of various strategies being used (e.g. if you hold an event and no one shows up, consider possible reasons why before your next event)
- Holding periodic planning meetings and updates with the program team
- Troubleshooting issues as they occur
- Brainstorming solutions to barriers that you may not have thought of during the planning phases

A key component of the monitoring process is ensuring that a standardized approach is used to calculate healthcare personnel immunization rates. Organizations repeatedly say this is one of the biggest challenges they encounter. See Appendix 2 for more information calculating rates.

While it is important to calculate healthcare personnel immunization rates consistently from year to year, it is of the utmost importance to take the time to review what was collected in the past and make the required changes to your data collection system.

Step 5 Evaluate and Celebrate

Once the influenza immunization campaign is over, it is time to reflect, evaluate, and celebrate.

To understand what worked well and what changes should be made to ensure future successes, you will have to evaluate what you did. You can choose to do a comprehensive evaluation, or pick one area of focus. Availability of resources, reporting requirements, and willingness of personnel to participate in the evaluation process will influence how comprehensive your evaluation will be.

When looking back on your program, it is important to determine whether your program met the objectives that were identified in Step 2 and to evaluate the process your team followed.

Process evaluation looks at the effectiveness of your program process.

Outcome evaluation focuses on the impact of your immunization program.

To conduct a process evaluation, ask your program team members to evaluate how they think they did based on the 5 Steps to Planning an Effective Influenza Immunization Program.

Example of a Process Evaluation

Step	Selected Measurement Indicator	Met	Unmet
1. Identify and Engage Your Program Team	Engage an epidemiologist to assist the team to determine what information we would like to collect , monitor, report, and evaluate	X	
2. Outline Your Implementation Plan	Review and update our HCP influenza immunization policy	X	
3. Determine Appropriate Components and Relevant Tools	Introduce at least two strategies that will allow for easier access to immunization for healthcare personnel	X	
4. Secure Resources, Implement, and Monitor	Obtain in-kind resources and support from neighbouring health centre		X
5. Evaluate and Celebrate	Host an event within two weeks after the campaign to share results and thank everybody who supported the program	X	

Explanation of an unmet indicator:

Step 4 – Timing did not allow for the development of a partnership. Beginning discussions have occurred with the neighbouring health centre in hopes of being able to work collaboratively next year.

To conduct an Outcome Evaluation you will need to use the data you collected during the program in order to determine if you achieved the objective(s) that were outlined in Step 2 when you identified your implementation plan.

Example of an Outcome Evaluation

If your goal was to increase the uptake of influenza immunization and your objective was to include three components – education, testimonials from leaders, and mobile carts – to increase the uptake of influenza immunization by 10% this year, your measurement indicator would be to determine whether the three components were included and whether immunization uptake increased by 10% this year.

A sample evaluation report based on this example would look something like this:

Goal	Objectives
To increase the uptake of influenza immunization	To include three components: education, testimonials from leaders, and mobile carts to increase the uptake of seasonal influenza immunization by 10% this year

Measurement Indicator	Met	Unmet
1. The 3 components (education, testimonials from leaders, and mobile carts) were included in this year's program	X	
2. Seasonal immunization uptake increased by 10% this year		X

Explanation of an unmet indicator:

- Objective 2 was not met because the dates conflicted with a staff reorganization that took priority.

What could your team have done differently (lessons learned)?

- We could have changed the dates for the seasonal influenza campaign, met with the senior VP to identify the impact the staff reorganization would have on the seasonal influenza campaign, and asked the VP to delay the reorganization until after the seasonal influenza campaign ended.

Additional examples to guide your outcome evaluation efforts can be found in Appendix 5 and a step-by-step approach to evaluation can be found in Appendix 6.

Remember to communicate your findings as soon as possible and celebrate milestones!

Conclusion

Successful programs are multifaceted and have commitment from organizational personnel at every level. They are continuously monitored, improved, and adapted using new knowledge and practices to increase healthcare personnel immunization rates.

While "it takes a village to raise a child" (African proverb), it takes a whole organization, with support at every level, to plan and implement a successful influenza immunization program.

Successful programs use a systematic approach and adopt multifaceted implementation strategies.

Appendix 1 ▶ Summary of Research Findings

We systematically reviewed the literature to identify studies that evaluated interventions aiming to increase influenza immunization coverage among healthcare personnel. Among the studies reviewed, programs involving only education or promotion resulted in small increases in vaccination rates relative to other interventions. In long-term care homes, programs with a greater variety of components (including education / promotion, better access to vaccines, legislation / regulation, and role models) were associated with higher immunization coverage. Coverage was highest when each worker had a personal interview session with a member of the study team. Within hospital settings, the results reported for various types of programs were mixed. Programs involving legislative or regulatory components (e.g. mandatory declination form, mandatory masks for unvaccinated personnel) achieved higher rates than other interventions. The major shortcoming of the reviewed studies was inadequate program evaluation. As a result, there was limited evidence on the effectiveness of program strategies.

Please consult the article published in the Canadian Medical Association Journal (11) to review the results in detail.

A systematic review conducted in 2008, and updated in 2009 (11), identified that:

- Regardless of the healthcare setting, programs with only an educational / promotional component resulted in minimal change in vaccine uptake. (Programs with more than one component were more effective in increasing immunization coverage.)
- Interventions with strategies directed at identified barriers were more effective than non-targeted strategies.
- The implementation of a new intervention is more successful when stakeholders and target groups are involved.
- Experience has shown that successful immunization programs engage advocates at all levels.
- Some components that have not been evaluated as extensively as others show promising results, but their effectiveness is unclear and further evaluation is needed (e.g. measurement / feedback, legislation / regulation and role models).
- Programs that included education / promotion and improved access resulted in an increased immunization rate of 25% in long-term care settings, whereas no increase was seen in hospital settings.
- Programs that focused on legislation / regulation resulted in an increased immunization rate of 15% in hospital settings.

The following activities were associated with improved HCP immunization rates:

- Traveling to different departments and units to offer the influenza vaccine
- Using pandemic preparedness strategies and initiatives to encourage workers to get the vaccine
- Offering incentives for receiving the influenza vaccine
- Encouraging workers to get immunized in order to avoid reassignment to another unit or shift during an influenza outbreak
- Organizing activities that encourage immunization and involve leaders and / or senior personnel
- Sharing immunization rates with personnel
- Using staff meetings as an opportunity to encourage immunizations

Appendix 2 ▶ Calculating Rates

Calculating accurate healthcare personnel influenza immunization rates is challenging for most healthcare organizations. Who do you count? Do you only count the people on payroll? What about physicians? What about volunteers? Who is responsible for providing accurate information? Do you require personnel immunized outside of the organization to provide written documentation? It is a challenging area and we cannot yet provide the best answer.

In a national survey in 2011, conducted in partnership with the Public Health Agency of Canada/ Canadian Institutes of Health Research Influenza Research Network Vaccine Coverage Working Group (PCIRN for short), we identified five categories that will capture most people providing service in the organization.

These categories are:

- Personnel on payroll (including casual personnel)
- Physicians not on your organization's payroll
- Personnel paid by an outside employer and not on your organization's payroll (i.e., contracted personnel, consultants)
- Medical residents, fellows and students, and students in other health (for example, nursing, laboratory technology) and health–related (e.g. social work, communication) disciplines
- Volunteers

It is also important to be able to identify personnel who are on the payroll, but are on extended leave (for example: maternity, sick or disability leaves), because they would not be expected to be immunized at work.

Clearly, not all organizations have personnel who fit into each category, but it does provide a starting place.

It is important to remember that whatever method is used to determine the numerator and denominator is used consistently from year-to-year. It is also important to record each year how you arrived at your final rate to allow for year-to-year comparisons.

Determining the Numerator

Having a good system for determining who the organization immunizes each year is normally straightforward. However, most organizations do immunize people who should not be included in the numerator as they are not included in the denominator. For instance, immunizing agency nursing personnel or contractors who work in your hospital is important to provide protection to your patients and other staff. However, you cannot count them in your numerator if you don't know the total number of agency staff or contractors who work in your organization.

One important challenge for all organizations is requiring personnel in each denominator category you report on to be required to provide written documentation of their immunization status. Check out the Toolkit for a Decision Form (www.chiin.ca, Step 2) that might be of benefit in assessing the

immunization intent of personnel before influenza season begins, or can be used as a reporting form for personnel who are immunized elsewhere.

Determining the Denominator

This is consistently problematic across organizations. Ideally, you will be able to know on any given day who is providing service to your patients or residents, but we know that most organizations cannot do that yet. You should work with your teams to build the most robust system possible.

Establish early on:

a) Who you can track with the system you have now. Most organizations have a way to track who is on payroll. In larger organizations, you may need to work with your information technology representatives to integrate the multiple databases available to you. Remember that your payroll database changes regularly with new hires and resignations. To help determine a reasonable denominator, you may want to set as your denominator people who were on staff on a particular day one week before the campaign starts.

b) Accounting for casual personnel is a challenge for many organizations. Casual personnel may work in multiple healthcare organizations and may be immunized elsewhere. Some may not work at your organization at all during the influenza season while others work regular hours. You should decide early on in your campaign how to include casual staff in your rates and how to ensure they are captured (or excluded) in both the numerator and denominator.

c) Determine who your organization considers as the people who should be immunized and work towards having a system to track those people. In larger organizations, you won't be able to track everyone (for example, medical students, or other students who stay at one hospital for relatively brief periods) without a regional system in place. Work with your senior leaders and networks to make this a reality.

d) Establish a method for setting your denominator. If you do not have a robust system where you can compare numerator and denominator data daily or weekly, then pick a date to set your denominator. For example, you can use the date your organization receives your first shipment of vaccine as the date you set your denominator.

e) Talk with the people or agencies (including public health) that you report to, and get their help in determining who should be included in the denominator and how they would like you to track the information.

f) Write it down. It is important to write down how you determined your numerator and denominator every year so you have a way to compare rates across time.

The tracking of healthcare personnel influenza immunization rates is important and is going to become even more important in the future. More and more, organizations will be asked to publically report their rates and healthcare personnel influenza immunization rates will be seen as a patient safety and quality indicator. Make sure you take the time in your planning period to address this important issue.

Key principal to considers

You cannot evaluate or change what you have not measured properly. In order to have confidence in your immunization rate, you must be able to calculate your rate based on the total number of healthcare personnel within your organization. Appendix 3 provides an example of one method for calculating the rates, by ensuring you can identify personnel who have the most patient contact.

It is important to use a standardized approach when calculating healthcare personnel immunization rates.

Appendix 3 ▶ Patient Contact Categories

Once you have determined the total number of HCP within your organization, you may want to consider determining who in your organization fits in each of these patient contact categories. Once you have this information, you can count how many HCP there are in each category. This will allow you to tailor your program objectives and components to those most at risk for getting or transmitting the influenza virus (12).

Table 1 – Patient Contact Categories (12)

Patient Contact Category	Definition	Examples
I	Personnel who have regular, close, face-to-face, hands-on contact with patients or residents as a part of a typical work shift, specifically including healthcare personnel who are regularly within 6* feet of a patient for ≥ 5 minutes	Physicians, clinical nurses, phlebotomists, medical assistants, personal support workers, radiology technicians, students in any of these categories, volunteers**, etc.
II	Personnel who have regular, day-to-day, face-to-face (but not hands-on) contact with patients	Front desk receptionist, psychiatrists, resource specialists, interpreters, transporters, volunteers**
III	Personnel who regularly provide services in patient care areas	Unit coordinators, patient liaisons, registrars, security, nutrition and environmental services personnel, volunteers**
IV	Personnel who have no patient contact, but perform functions vital to clinical service delivery	Personnel who regularly handle specimens for testing and diagnostics
V	Personnel who do not have patient contact	Personnel in human resources and fiscal departments, volunteers**

* We changed the number of feet to reflect the new guidelines published by the Ministry of Health and Long-Term Care in Ontario (17)

** A volunteer's level of patient contact will vary, depending on his/her role and responsibilities in the healthcare organization

Knowing the uptake rate of personnel by category will enable you to tailor your program the following year to reach those individuals you did not reach this year.

Appendix 4 ▶ The Ottawa Influenza Decision Aid

What Is the Ottawa Influenza Decision Aid (OIDA)?

The OIDA is a decision aid that was developed in 2006 by the Canadian Healthcare Influenza Immunization Network (CHIIN) team (10). It was created to address the suboptimal influenza vaccine uptake rate among healthcare personnel. The OIDA was designed to lower decisional conflict and help personnel make an informed decision about influenza prevention and immunization.

The OIDA is a four-page pamphlet available in both French and English. It presents research evidence in text; image and number format; questions about preferences, values, knowledge, and support; and the benefits and side effects of each option related to influenza immunization. It takes an individual through the steps of decision-making with a balanced, clear, and easy-to-read layout. It was developed and based on the Ottawa Decision Support Framework (OPDS) and adjusted to meet the International Patient Decision Aid Standards (http://ipdas.ohri.ca). A copy of the OIDA can be obtained online at www.chiin.ca.

Why Use the OIDA in Your Program?

The annual decision about influenza immunization may be a difficult one for some healthcare personnel to make. Decisional conflict may be a contributing factor to poor vaccine uptake and could result in individuals remaining undecided (15). The OIDA is aimed at increasing knowledge and clarifying the personal values of healthcare personnel with regards to influenza prevention options and decision making.

The OIDA can be tailored to reflect your organization's influenza vaccine policy, which makes it a useful tool within your organization's annual influenza immunization program. It is designed to be used as one of the tools in your multi-intervention program.

Optimal ways to use the OIDA are currently being explored. Options include an interactive web tool, as well as a hard copy. Preliminary evidence suggests it is most effective when used one-on-one as part of a small group educational session. This type of intervention allows personnel to discuss the choices they have in the context of their values, organizational policy, scientific evidence, and specific knowledge about the influenza vaccine.

The OIDA is a tool that can respond to your organization's unique culture as you optimize your existing immunization program in order to achieve the recommended 100% immunization uptake rate (18). The hope is that, with the implementation of new tools such as the OIDA, the vaccine uptake rates will improve in the interpandemic period as your organization strives toward an improved infrastructure that supports the national and World Health Organization global pandemic preparedness goals (18, 19).

Examples of how to use the Ottawa Influenza Decision Aid in your program:

- Promote the OIDA at workplace educational fairs / displays
- Use the OIDA in a group setting, such as regular staff meetings
- Use the OIDA as a one-on-one consultation / educational tool with undecided employees
- Combine the OIDA with an influenza immunization consent form
- Have a designated person implement the OIDA with personnel

Appendix 5 ▶ Outcome Evaluation Examples

Goal
To calculate accurate influenza immunization rates for our organization

Objective 1		
Our program team will establish a systematic way to count the number of paid and unpaid personnel for this year's program.		
**Measurement Indicator**	**Met**	**Unmet**
We established a systematic way to count the number of paid and unpaid personnel for this year's program.		

Objective 2		
Our program team will establish a comprehensive method to determine who was and who was not immunized this year so year-to-year comparisons can be made.		
**Measurement Indicator**	**Met**	**Unmet**
We established a comprehensive method to determine who was and who was not immunized this year.		

Goal
To communicate the influenza immunization rates at selected intervals to the appropriate informal and formal entities/bodies.

Objective 1		
Our program team will report immunization rates at regular intervals to staff, managers, and senior administrators during and after this year's campaign.		
**Measurement Indicator**	**Met**	**Unmet**
Immunization rates were communicated at regular intervals to staff, managers, and senior administrators during the program.		
Immunization rates were communicated to staff, managers, and senior administrators at the end of the program.		

Objective 2		
To comply with formal reporting requirements of public and government agencies.		
**Measurement Indicator**	**Met**	**Unmet**
Our program team complied with the formal reporting requirements of public and government agencies.		

Document the milestones that were met along with the explanations for the milestones you were not able to meet.

Appendix 6 ▶ Step-by-Step Approach to Evaluation

1. Decide specifically what you want to evaluate. Do you want to evaluate what worked well or what didn't work well? How about the policy? Did it work? Did you have tangible support of senior management? What are your reporting requirements?

2. Determine what support you need to complete your evaluation. If you need the support of line managers, which decision-maker will allow you to contact them and will ensure compliance with your efforts? Make sure you involve the decision-makers in the planning.

3. Select the type of approach you want to use to gather your information. For example:
   - Face-to-face meeting with an open-ended discussion
   - Face-to-face meeting with defined questions
   - Electronic survey (using tools like "Survey Monkey" or "Remark")
   - Paper survey (to use this method, you need to determine distribution techniques, i.e. mail to home, distribute at work, attach to pay stub)
   - Decide on your questions

4. Determine how you will do your analysis. Is there someone with experience in evaluation analysis? Can you contact your research institute for support if you have one? How much detail do you need?

5. Determine whether you will provide incentives to encourage people to participate in discussions or to fill out surveys. Make sure the incentives are appropriate and approved by the necessary decision makers.

6. Conduct your evaluation. Make sure you include time to follow up with people who have not responded.

7. Complete your analysis.

8. Use the results to make improvements to your program for the next year.

Appendix 7 ▶ Seasonal Influenza Immunization Planning Calendar Template

Month	Activity	Person Responsible	Date Completed
**Assemble a multidisciplinary program team**			
	Identify your multi-disciplinary team using the Human Resources Planning Tool. This team will be responsible for the planning, implementation and evaluation of the immunization campaign.		
February - March	Convene a multidisciplinary team meeting to complete the Pre-Program Assessment Checklist.		
	Designate team members who will:<br>▪ Seek and obtain support from opinion leaders and senior managers. (Consider using the Letter to Elicit Support from Senior Management that is in the toolkit)<br>▪ Coordinate the marketing and communications functions<br>▪ Develop a broad communication plan<br>▪ Assist in determining what information should be collected and determine how the immunization rates will be calculated		
**Outline your implementation plan**			
February - March	Review the evaluation from the previous year's influenza program:<br>▪ Identify strengths, weaknesses, opportunities and threats<br>▪ Identify potential barriers to implementation and immunization<br>▪ Review how many healthcare personnel were immunized last year<br>▪ Set a target immunization rate for this year's campaign<br>▪ Identify your campaign goals and objectives (Use SMART objectives)		
	Engage individuals who normally are not immunized to assist in identifying potential solutions to the barriers identified.		

**Determine campaign tools**			
	Review your healthcare personnel immunization policy for seasonal influenza immunization:<br>▪ Create or revise as needed<br>▪ Consider the inclusion of an immunization decision form (e.g. Consent/declination form)		
March	Consider adopting some of the implementation tools that are available.		
	Work with your committee to develop a logistical plan:<br>▪ Plan multiple clinics, easy access, flexible hours etc,<br>▪ Plan a "launch" event<br>▪ Confirm that you have institutional support and commitment from senior management (Consider using the Letter to Elicit Senior Management Support in the toolkit)<br>▪ Finalize your communication plan		
**Secure Resources, Implement and Monitor**			
May	Identify the human resources you will be need:<br>▪ Confirm that you have access to committed and well-informed individuals available to immunize		
	Develop a budget that identifies the financial resources needed for your influenza immunization program:<br>▪ Secure the resources you need		
	Gather educational materials and develop a staff presentation that can be used by team managers.		
June to July	Plan a promotional event and secure resources to promote and launch the campaign:<br>▪ Include activities that encourage immunization and involve leaders and/or senior staff<br>▪ Choose incentives to sway the undecided (i.e. Stickers, time off, quizzes, contests, prizes, etc)<br>▪ Develop posters, buttons etc		
	Establish a system to track and monitor results:<br>▪ Identify a process to calculate and compare your immunization rates		

	Develop an evaluation plan.		
	Meet with your team to finalize your influenza immunization program plan.		
	Begin promoting your campaign via e-mail, posters, flyers, newsletter articles, pay stubs, flyers.		
	Confirm the timing of influenza vaccine and place the order.		
August	Provide training for additional nursing staff to administer influenza vaccine within their departments.		
	Obtain recent information from provincial health authorities, the Public Health Agency of Canada, National Advisory Committee on Immunization, Immunize Canada or other relevant site.		
	For the upcoming campaign:<br>▪ Obtain frontline manager support<br>▪ Obtain medical director / physician support<br>▪ Consider using the letter to Elicit Support from Managers that is in the toolkit<br>▪ Consider using the letter to Elicit Support from physicians in the toolkit		
	Identify important meetings and events where the campaign can be promoted.		
September	Launch the campaign and include senior managers and organizational leaders in the launch.		
	Initiate the seasonal influenza promotional campaign activities:<br>▪ Send ads and put up posters<br>▪ Make reference material accessible for staff who may want to learn more about influenza (inter/intranet sites, publications, etc)<br>▪ Offer healthcare personnel education sessions		
	Continue to promote and encourage influenza immunization.		

October	Implement your healthcare personnel immunization campaign.		
	Monitor immunization rates.		
	Communicate immunization rates to staff and management.		
November	Continue to promote and encourage influenza immunization.		
	Monitor immunization rates.		
December	Continue to promote and encourage influenza immunization.		
**Evaluate and Celebrate**			
January	Assemble the team to evaluate the immunization program:<br>▪ Identify areas of success and areas for improvement<br>▪ Compile immunization statistics<br>▪ Make sure you advise your senior leadership team of the successes of your program. This will help build support for changes you identify from your evaluation.<br>▪ Work with your communications representatives to ensure that the challenges and successes are shared with all personnel, as well as with senior leaders. Don't forget to include the Board of Directors of your organization when communicating the results of your campaign and overall program. You will need to work with your communications and senior leader representatives to achieve this.		
	Implement a post-immunization campaign event / strategy to thank everyone and to share the results with everyone in the organization:<br>▪ Disseminate the campaign results<br>▪ Recognize champion units or departments<br>▪ Congratulate participants for their contributions to the success of the campaign		

Other Considerations

Consider adding to the items to your calendar. These are dependent on your organization's requirement for timing of requests:

- Determining anticipated amounts of supplies needed
- Ordering Supplies
- Deadline dates for communications items:
    - Printing
    - Advertisements
    - Inserts for check stubs etc
    - Time frames for announcements and launch
- Operational items:
    - Room bookings
    - Human resource posting and recruitment deadlines

Adapted from:

APIC Toolkit located at http://www.apic.org/Content/NavigationMenu/PracticeGuidance/Topics/Influenza/toolkit_welcome.htm

ASSTSAS Trousse Influenza 2009-08-25 Located at http://www.asstsas.qc.ca/francais/information_et_conseil_en_sst/dossiers_thematiques/virus_de_la_grippe/dossier_infections___trousse_influenza_2009.html

References

1. Public Health Agency of Canada. Guide to Project Evaluation: A Participatory Approach [Internet]. 2001 [updated 2001 Dec 8; cited 2010 Jul 30]. Available from: http://www.phac-aspc.gc.ca/ph-sp/resources-ressources/guide/defining-eng.php.

2. Accreditation Canada. Patient Safety Area 5: Infection Control [Internet]. 2009 [cited 2010 Jul 30]. Available from: http://www.accreditation.ca/uploadedFiles/flu%20vaccine.pdf?n=3070.

3. Harper SA, Fukuda K, Uyeki TM, Cox NJ, Bridges CB. Prevention and control of influenza: recommendations of the advisory committee on immunization practices (ACIP). Morbidity and Mortality Weekly Report. Recommendations and Reports. 2004; 53(RR-6): 1-40.

4. Potter J, Stott DJ, Roberts MA, Elder AG, O'Donnell B, Knight PV. Influenza vaccination of healthcare workers in long-term-care hospitals reduces the mortality of elderly patients. J Infect Dis. 1997; 175(1): 1-6.

5. Carman WF, Elder AG, Wallace LA, McAulay K, Walker A, Murray GD, Stott DJ. Effects of influenza vaccination of health-care workers on mortality of elderly people in long-term care: a randomised controlled trial. Lancet. 2000 Jan 8;355(9198):93-7.

6. Hayward AC, Harling R, Wetten S, Johnson AM, Munro S, Smedley J, Murad S, Watson JM. Effectiveness of an influenza vaccine programme for care home staff to prevent death, morbidity, and health service use among residents: cluster randomised controlled trial. BMJ. 2006 Dec 16;333(7581):1241. Epub 2006 Dec 1.

7. Lemaitre M, Meret T, Rothan-Tondeur M, Belmin J, Lejonc JL, Luquel L, Piette F, Salom M, Verny M, Vetel JM, Veyssier P, Carrat F. Effect of influenza vaccination of nursing home staff on mortality of residents: a cluster-randomized trial. J Am Geriatr Soc. 2009 Sep;57(9):1580-6. Epub 2009 Aug 4.

8. Cunney RJ, Bialachowski A, Thornley D, Smaill FM, Pennie RA. An outbreak of influenza A in a neonatal intensive care unit. Infect Control Hosp Epidemiol. 2000; 21(7): 449-454.

9. Pearson ML, Bridges CB, Harper SA. Influenza vaccination of healthcare personnel: recommendations of the healthcare infection control practices advisory committee (HICPAC) and the advisory committee on immunization practices (ACIP). Morbidity and Mortality Weekly Report. 2006; 55(RR-2): 1-16.

10. Chambers LW, Wilson K, Hawken S, Puxty J, Crowe L, Lam PP, Farmanova-Haynes E, McNeil SA, McCarthy AE. Impact of the Ottawa Influenza Decision Aid on healthcare personnel's influenza immunization decision: a randomized trial. J Hosp Infect. 2012 Nov;82(3):194-202. doi: 10.1016/j.jhin.2012.08.003. Epub 2012 Sep 24.

11. Lam P, Chambers LW, Pierrynowski MacDougall DM, McCarthy AE. Seasonal immunization programs for healthcare personnel: systematic review. Can Med Assoc J. 2010 Jul; doi:10.1503/cmaj.091304.

12. The Joint Commission. Providing a safer environment for Healthcare Personnel and Patients through Influenza Vaccination. U.S.A.: Joint Commission on Accreditation of Healthcare Organizations; 2009. vii p. Available from: http://www.jointcommission.org/assets/1/18/flu_monograph.pdf

13. Last JM, Spasoff RA, Harris SS. A Dictionary of Epidemiology. 4th ed. New York: Oxford University Press; 2001.

14. Natural Resources Canada. Guidelines In-kind Support [Internet]. 2009 [updated 2009 Apr 22; cited 2010 Jul 30]. Available from: http://oee.nrcan.gc.ca/transportation/afo/forms/guidelines-in-kind.cfm?attr=8

15. O'Connor AM, Bennett CL, Stacey D, Barry M, Col NF, Eden KB, Entwistle VA, Fiset V, Holmes-Rovner M, Khangura S, Llewellyn-Thomas H, Rovner D. Decision aids for people facing treatment or screening decisions. Cochrane Database of Systematic Reviews [Internet] 2009 [cited 20102 Nov 20]; 3: CD001431. Available from: http://onlinelibrary.wiley.com/doi/10.1002/14651858.CD001431.pub2/abstract

16. Public Health Agency of Canada. Guide to Project Evaluation: A Participatory Approach [Internet]. 2001 [updated 2001 Dec 8; cited 2012 Nov 20]. Available from: http://www.phac-aspc.gc.ca/ph-sp/resources-ressources/guide/index-eng.php

17. Ministry of Health and Long-Term Care: Provincial Infectious Diseases Advisory Committee (PIDAC). Best Practices For Infection Prevention and Control of Resistant Staphylococcus aureus and Enterococci [Internet]. 2007 Mar [cited 2012 Nov 20]. Available from: http://www.fields.utoronto.ca/programs/scientific/10-11/drugresistance/emergence/katz1.pdf

18. McCarthy A. Decision aid to increase vaccination among front-line healthcare workers. Can J Infect Dis Med. 2006; 17(6): 371.

19. Sullivan SM, McCarthy AE, Chambers LW, O'Connor A, Knoefel F, Sutherland J, Pierrynowski-McDougall D, Baker D, Villeneuve J, Arnold P. Development and evaluation of a decision aid about influenza prevention for healthcare workers. Can J Infect Control. 2007; 22(4): 203-210.

Canadian Healthcare
Influenza Immunization
Network

Réseau Canadien
d'Immunisation contre l'Influenza
dans les Soins de Santé
