# Supplementary material for: A New Approach to Improving Healthcare Personnel Influenza Immunization Programs: A Randomized Controlled Trial
Source: PLoS One. 2015 Mar 17;10(3):e0118368. doi: 10.1371/journal.pone.0118368 (PMC4363667; doi:10.1371/journal.pone.0118368)
Supplement: S2 File — (PDF) [file pone.0118368.s005.pdf]

Healthcare Personnel Seasonal Influenza Immunization Program
Human Resource Planning Tool (Step 1)

"A team comprises a group of people linked in a common purpose. Teams are especially appropriate for conducting tasks that are high in complexity and have many interdependent subtasks."[1]

It is important to establish links and partnerships with others in your organization who share the same mandate to protect and promote the wellbeing of healthcare personnel.

--

Who to Recruit?

- Occupational Health and Safety representative
- Chief Executive Officer or Senior Vice President
- Representatives from all levels and programs across the healthcare organization
- People who are qualified to administer the vaccine
- Gatekeepers and opinion leaders
- People who know how to maintain cold chain
- Pharmacy representative
- Communications representative
- Frontline / unit level personnel including personal support workers / care attendants
- Joint union and occupational health and safety committee member / union representatives
- Information technology representative
- Nursing team members
- Volunteer co-ordinators

Team Members Attributes to Look for:

- Advocates influenza immunization and is committed to increasing influenza immunization rates
- Able to leverage or commit support for campaign, including allocation of resources and ensuring support from all levels of management
- Is a positive role model
- Has experience in communications
- Understands how to effect change
- Is well-organized and able to keep on task
- Understands the importance of resolving union / related issues early in the process
- Able to transfer knowledge to information technology staff to facilitate the development of a user-friendly database that will produce useful reports
- Able to help the team prioritize activities

> When inviting individuals to participate, they should be made aware that their level of involvement will ebb and flow, depending on the stage of the program and they will only need to participate during those times when their input is required.

[1] http://en.wikipedia.org/wiki/Team

Worksheet

What roles do we need filled?	Who can fill the role?	Role allocated
**Core Program Team**		
Team Leader		☐
Administrative Support		☐
Senior Administration Sponsor		☐
Nursing Team Members		☐
Communications		☐
Pharmacy		
Information Technology Representative		☐
Epidemiologist or Statistician		☐
Others:		☐
		☐
		☐
		☐
		☐
**Other Roles**		
Allied Health and Frontline Support Workers		☐
Union Representatives / Workplace Health and Safety Committee Members		☐
Volunteers		☐
Others:		☐
		☐
		☐
		☐
		☐

Canadian Healthcare Influenza Immunization Network
Réseau Canadien d'Immunisation contre l'Influenza dans les Soins de Santé
www.chiin.ca

Healthcare Personnel Seasonal Influenza Immunization Program
Letter to Elicit Support from Senior Management (Step 1)

Date

Name
Address

Dear:

Re: Senior Management Support and Involvement in the Annual Healthcare Personnel Influenza Immunization Campaign

Experience and research has shown that successful immunization programs engage champions at all levels. Support from senior management, clinical leaders and opinion leaders have demonstrated positive outcomes and increased rates.

The best evidence points to the advantages of obtaining the highest possible healthcare personal influenza immunization rate, which is often quoted as 95% to 100%.

Despite abundant evidence about the safety and effectiveness of the influenza vaccine, last year our organization achieved an immunization rate of ___%.

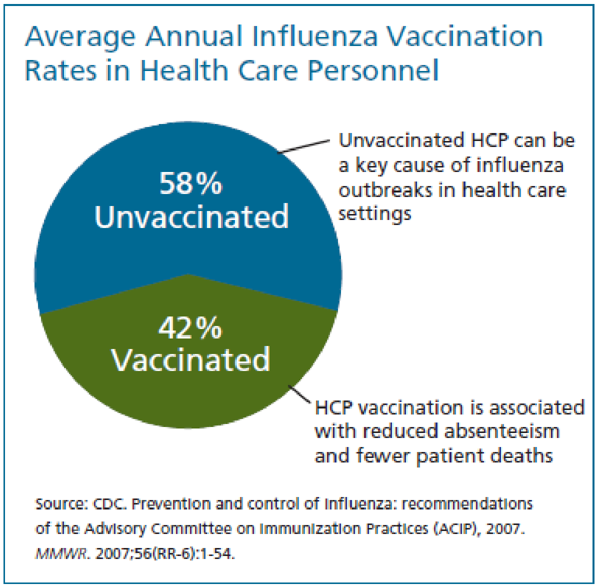

We need your support to assist with the promotion of our annual influenza immunization campaign.

Successful programs are multifaceted and require commitment from organizational staff at all levels. We are requesting the support of senior managers and leaders within our organization to assist with the promotion of our annual influenza immunization campaign. More information about specific details will follow, but examples of some of the activities may include: making a statement of priority for the campaign; email communiqués from the CEO/President or senior MOH; public appearances at large immunization clinics; attendance at the campaign launch; encouraging healthcare personnel immunization by highlighting the importance of influenza immunization at meetings and public gatherings; offering incentives, etc.

Respectfully,

Influenza Immunization Program Planning Committee Chair

Credit: Canadian Healthcare Influenza Immunization Network, 2012

Canadian Healthcare Influenza Immunization Network

Réseau Canadien d'Immunisation contre l'Influenza dans les Soins de Santé

www.chiin.ca

Healthcare Personnel Seasonal Influenza Immunization Program Planning Calendar (Step 1)

February to April Activities

Activity	Step	Person Responsible	Date Completed
1. Identify your multidisciplinary team using the Human Resources Planning Tool. This team will be responsible for the planning, implementation and evaluation of the immunization campaign.	1		
2. Convene a multidisciplinary team meeting to complete the Pre Program Assessment Checklist.	1		
3. Designate team members who will:<br><br>• Seek and obtain support from opinion leaders and senior managers (Consider using the Letter to Elicit Support from Senior Management that is in the Tool Kit)<br>• Coordinate the marketing and communications functions<br>• Develop a broad communication plan<br>• Assist in determining what information should be collected and determine how the immunization rates will be calculated	1		
4. Review the evaluation from the previous year's influenza program:<br><br>• Identify strengths, weaknesses, opportunities and threats<br>• Identify potential barriers to implementation and immunization<br>• Review how many healthcare personnel were immunized last year<br>• Set a target immunization rate for this year's campaign	2		
5. Engage individuals who normally are not immunized to assist in identifying potential solutions to the barriers identified.	2		

Credit: Canadian Healthcare Influenza Immunization Network, 2012

6. Review your healthcare personnel immunization policy for seasonal influenza immunization.<br><br>• Create or revise as needed.<br>• Consider the inclusion of an immunization decision form (e.g. consent/Decision Form).	2		
7. Identify new program goals and objectives (consider using SMART objectives).	2		
8. Consider adopting some of the implementation tools that are available in the Tool Kit.	3		
9. Work with your committee to develop a logistical plan:<br><br>• Plan multiple clinics, easy access, flexible hours etc. Plan to book clinic rooms in advance if possible. Consider alternate spots for clinics to increase visibility<br>• Plan a “launch” event<br>• Confirm that you have organizational support and commitment from senior management (Consider using the Letter to Elicit Senior Management Support in the toolkit)	3		
10. Develop your communications plan.	2, 3		

May Activities

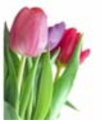

Activity	Step	Person Responsible	Date Completed
1. Meet with your team to discuss progress, identify priorities and resource issues.	4		
2. Identify the human resources you will be need.	4		
3. Confirm that you have access to committed and well-informed individuals available to immunize.	4		
4. Develop a budget that identifies the financial resources needed for your influenza immunization program<br>• Secure the resources you need<br>• Consider asking for reallocation of resources from other areas<br>• Remember to consider in-kind support requests	4		

June and July Activities

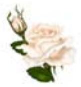

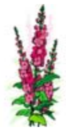

Activity	Step	Person Responsible	Date Completed
1. Gather educational materials and develop a staff presentation that can be used by team managers.	3		
2. Plan a promotional event and secure resources to promote and launch the campaign.<br><br>• Include activities that encourage immunization and involve leaders and/or senior staff<br>• Choose incentives to sway the undecided (i.e. Stickers, time off, quizzes, contests, prizes, etc)<br>• Develop posters, buttons, etc.	3		
3. Establish a system to track and monitor results<br><br>• Identify a process to calculate and compare your immunization rates<br>• Make sure you write down how you are planning to calculate your denominator and numerator, and make sure that you keep this record every year, so you can compare across time (see Appendix 2 for more details)	2		
4. Develop a comprehensive evaluation plan.	2		
5. Review all your planning to-date and identify any pieces that are missing, and find a way to address those issues (continually assess and reassess, take advantage of new opportunities, adjust your plan as things in your organization change).	1-5		

August Activities 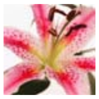

Activity	Step	Person Responsible	Date Completed
1. Meet with your team to finalize your influenza immunization plan.	1		
2. Begin promoting your campaign via e-mail, posters, flyers, newsletter articles, pay stubs.	3		
3. Confirm the timing of influenza vaccine and place the order.	4		
4. Provide training for additional nursing staff to administer influenza immunizations within their departments. Consider training influenza champions.	4		
5. Obtain recent information from your direct vaccine supplier to confirm shipment dates for the vaccine.			
6. Review team membership – now is when you need to invite everyone to move into higher gear. Do you have all the support you need? Who else do you need to bring on board? Have a larger team meeting to bring everyone together to remind people of the campaign, and their commitments.	4		

September and October Activities

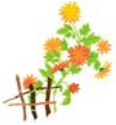

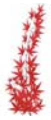

Activity	Step	Person Responsible	Date Completed
1. Identify important meetings and events where the campaign can be promoted.	4		
2. Launch the campaign and include management/leaders and senior staff in launch (timing will depend on vaccine availability).	4		
3. Initiate the seasonal influenza promotional campaign activities :<br>• send ads and put up posters<br>• make reference material accessible for staff who may want to learn more about influenza (inter/intranet sites, publications, etc)<br>• offer healthcare personnel education sessions	4		
3. Continue to promote and encourage influenza immunization.	4		
4. Implement your healthcare personnel immunization campaign.	4		
5. Monitor immunization rates. Evaluate what you are doing and change as necessary.	4		
6. Report immunization rates to staff, managers, senior leadership team, patients/residents and volunteers.	4		

November and December Activities

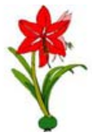
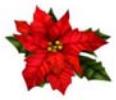

Activity	Step	Person Responsible	Date Completed
1. Continue to promote and encourage influenza immunization.	4		
2. Continue monitoring and reporting immunization rates.	4		
3. Continue to work with your team, provide encouragement, adjust planning if needed, consider unique ways to give your campaign a boost once the initial phase is over.	4		

January Activities

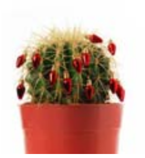

Activity	Step	Person Responsible	Date Completed
1. Assemble the team to evaluate the immunization program.<br><br>• Identify areas of success and areas for improvement<br>• Compile immunization statistics and report them to everyone	5		
2. Implement a post-immunization campaign event / strategy to share the results with healthcare personnel and senior leaders.<br><br>• Disseminate the campaign results. Think about including successes beyond reporting rates<br>• Recognize champion units or departments<br>• Congratulate participants for their contributions to the success of the campaign	5		
3. Continue to immunize as needed.	5		
4. Congratulate yourself on managing another challenging program! Celebrate those successes. Build-up goodwill for next year.	5		

Other Considerations

Consider adding to the items to your calendar (dependent on organizational time frames):

- Determining anticipated amounts of supplies needed
- Ordering Supplies
- Deadline dates for communications items
    - Printing
    - Advertisements
    - Inserts for check stubs etc.
    - Time frames for announcements and launch
- Operational items
    - Room bookings
    - Human resource posting and recruitment deadlines

Adapted from:

APIC Toolkit located at: http://www.apic.org/Content/NavigationMenu/PracticeGuidance/Topics/Influenza/toolkit_welcome.htm
ASSTSAS Trousse Influenza 2009-08-25 Located at:
http://www.asstsas.qc.ca/francais/information_et_conseil_en_sst/dossiers_thematiques/virus_de_la_grippe/dossier_infections___trousse_influenza_2009.html

Canadian Healthcare Influenza Immunization Network
Réseau Canadien d'Immunisation contre l'Influenza dans les Soins de Santé
www.chiin.ca

Healthcare Personnel Seasonal Influenza Immunization Program
Pre-Campaign Assessment Worksheet (Step 1)

Step 1

Assessment of Current Program	Yes	No	New Goals and Objectives
1. Did you use a Post-Campaign Assessment Worksheet last year?<br><br>• If yes, have you reviewed the recommendations from last year?			
2. How many healthcare personnel were immunized last year? _________			
2. Have you established a multidisciplinary planning team?			
3. Have you included opinion leaders and senior managers?			
4. Have you designated a team member to coordinate the marketing and communications functions?			
5. Last year, which departments or disciplines had the least number of staff members receiving their influenza immunization? (e.g. physicians, nurses, housekeepers, maintenance personnel etc.)?<br><br>____________________________________<br>____________________________________<br>____________________________________<br>____________________________________			
6. Have you invited representatives from these groups to participate on the planning committee?			

Step 2

Assessment of Current Program	Yes	No	New Goals and Objectives
1. Will you require personnel to report their immunization status?			
2. Will you require personnel to sign a form if they choose not to be immunized (decision/consent form)?			
3. Will you use immunization champions or role models?<br>• At the senior management level?<br>• At the management level?<br>• At the unit / staff level?			

Step 3

Assessment of Current Program	Yes	No	New Goals and Objectives
1. What methods will you use to improve immunization access?<br><br>• Kick-off<br>• On-site clinic<br>• Rolling cart clinics<br>• Other: ______________________			
2. What tools will you use for campaign promotion and staff education?<br><br>• Flyers/handouts<br>• Posters<br>• Email<br>• Employee newsletters<br>• Articles from newspapers / publications / on-line sites<br>• Information attached to pay stubs before campaign / during campaign / post campaign<br>• In-service training<br>• Other: ____________________			
3. What incentives or rewards will you use to engage the undecided?<br><br>• Departmental competitions<br>• Refreshments<br>• Raffle<br>• Games<br>• Other: _______________________			
4. Have you established an evaluation framework for your immunization program?			
5. Have you done a SWOT analysis?<br><br>• Strengths<br>• Weaknesses<br>• Opportunities<br>• Threats			

Credit: Canadian Healthcare Influenza Immunization Network, 2012

Step 4

Assessment of Current Program	Yes	No	New Goals and Objectives
1. Have you established a method to track your immunization progress?			
2. Will you be able to reflect your numbers using a rate?			
3. Will you be able to track all staff?			
4. Is the tracking system flexible?			
5. Have you considered how to identify and secure the human and financial resources required?			

Step 5

Assessment of Current Program	Yes	No	New Goals and Objectives
1. Do you have a plan to share the immunization rates with the following groups?<br><br>• Staff<br>• Senior leadership team<br>• Board of directors<br>• Community			
2. Are you planning a post-campaign event to celebrate successes?			
3. How are you planning to thank members of your influenza prevention team?			

Healthcare Personnel Seasonal Influenza Immunization Program
Considerations for Developing a Healthcare Personnel Immunization Policy (Step 2)

What is a Policy?

A policy is a set of guiding principles, an acceptable practice, or a rule intended to influence organization decision making. Policies typically support management philosophies and help to communicate regulations that apply to all personnel. Policies are formal in nature, broad in their application, and rarely change unless a regulation, law, or code of practice changes in the industry.[1]

Policies can differ in their format and design from one organization to another however at minimum a policy should:

- Reflect the organization's mission and goals
- Be expressed as a broad statement
- Include statements of "what" and/or "why"
- Address major operational issues
- Be clear and concise
- Meet current regulations, laws and best practices (may reference specific laws, regulations, or codes of practice in the body of the policy if necessary)

Policies reflect the "rules" governing the implementation of the organization's processes.[2]
The overall goal for any policy is for the design to be simple, consistent, and easy to use.

Writing Tips

- Policies should be clear, concise and use simple language.
- Polices should be factual and accurate.
- Policies should avoid the use of acronyms. If you choose to use acronyms, be sure to spell them out the first time you use them in the document.
- Policies must be simple enough to be understood by all employees.

Key Points in Developing a Good Policy

- Policy statements address what the rule is rather than how to implement the rule.
- Policy statements should be readily available to all healthcare personnel and their authority should be clear.
- Policies represent a consistent, logical framework for action.
- Policies shouldn't include information that may be quickly outdated (e.g., instead of referencing names, reference positions).

Key Points Specific to Influenza Immunization Policies

- Indicate who should be immunized, why, and when.
- Include a detailed goal, such as attaining a 95% immunization rate.
- Consider your organizational culture. (For example if unions or employees are uncomfortable with provisions in the policies, listen to their concerns and respond appropriately.)
- Determine the most appropriate policy for your type of organization as policies may differ between organizations.
- If feasible, extend the policy to all visitors of the facility.

Implementation and Dissemination Tips

- Use institutional methods for policy approval.
- Make the policy available to all employees and contract workers.
- Designate "policy experts" (identified in each document) to interpret the policy and resolve issues.
- Use your policy dissemination strategy as an opportunity to educate leadership and staff about the importance of getting immunized.

---

[1] Council on Accreditation,8th Edition Standards, June 2008
[2] Policy Coordinating Office, Policy and Procedures Team, Internal Working Draft , Printed 12/2/1994

Canadian Healthcare Influenza Immunization Network
Réseau Canadien d'Immunisation contre l'Influenza dans les Soins de Santé
www.chiin.ca

Healthcare Personnel Seasonal Influenza Immunization Campaign
Influenza Immunization Policy Sample (Step 2)

Note: Some phrases will need to be personalized based on terminology of the respective organization.

Preamble

In the spirit of "doing no harm" and to meet expectations of a patient-safety oriented healthcare system, all healthcare personnel have a responsibility to take all reasonable action to avoid influenza transmission to those in their care. Healthcare personnel are in a unique position to act as carriers for influenza whether or not symptoms are present. Patients / residents are vulnerable and at high risk of developing serious influenza-related complications that can be fatal.

Immunizing healthcare personnel (and patients / residents) is a proven and efficient preventative measure to prevent the spread of influenza, influenza outbreaks and related deaths. The influenza vaccine is recommended for all healthcare personnel, unless medically contraindicated.

Goal

The goal of this policy is to support the organization to reach and maintain the highest possible healthcare personnel influenza immunization rates, which is often quoted as 95% - 100%.

Policy

<insert name of organization> is using evidence informed practices related to improving influenza immunization rates of healthcare personnel. This policy applies to all persons who carry on activities in this organization including; employees, students, medical residents, physicians, volunteers, pastoral care and contract workers (or collectively "healthcare personnel", see definition below).

The organization will:

- offer healthcare personnel a complete and proactive influenza immunization program
- provide annual education on influenza and the vaccine
- provide free influenza immunization to all healthcare personnel through a series of published on-site clinics, to prevent the occurrence and spread of influenza
- provide information to all new hires regarding the need for annual influenza immunization
- outline the organizations' expectation that all will adhere to the influenza immunization policy at the time of hiring
- provide all new hires a copy of both the Influenza Immunization Policy and the Seasonal Influenza Immunization Decision Form
- ensure that all healthcare personnel adhere to the organization's influenza immunization policy
- will ask for proof of influenza immunization if the hiring falls during influenza season (November to April)

- offer influenza immunization to healthcare personnel not yet immunized
- inform outside staffing agencies and schools that they are responsible for adherence to the influenza immunization policy (appropriate education, immunization and maintenance of immunization records for their healthcare personnel)
- ask healthcare personnel who have been immunized elsewhere to provide written proof of immunization
- ask healthcare personnel who claim to have a medical contraindication to provide physician documentation (see valid medical exemption in definitions below)
- will provide managers with a list of the immunization status of their direct reports

Healthcare personnel are responsible for:

- making an informed decision by reviewing the educational materials
- completing the Seasonal Influenza Immunization Decision Form annually and submitting it to their manager/supervisor/program lead by the designated date identified by the organization
- keeping an updated record of immunization and providing a copy of their proof of immunization when requested by the organization
- knowing that if they choose not to be immunized, there may be repercussions (such as work reassignment, wearing personal protective equipment) in the event of an influenza outbreak, as outlined in the organization's influenza outbreak policy

Department / division heads are responsible for:

- ensuring that all medical staff and residents adhere to the organization's influenza immunization policy

Definitions

Healthcare personnel

The term healthcare personnel (HCP) is defined broadly as all paid and unpaid persons working in healthcare settings.

Healthcare organization (or setting)

Healthcare personnel work in a variety of settings, including (but not limited to) acute care hospitals, long-term care facilities, skilled nursing facilities, rehabilitation centres, urgent care centres, outpatient clinics, home and community healthcare agencies, and emergency medical services.

Valid medical exemptions to influenza immunization

- persons with a history of an anaphylactic reaction to a previous dose of the vaccine or to any component of the vaccine
- persons with a history of Guillain-Barré Syndrome

For more information about this policy, please contact <name and contact information>.

Healthcare Personnel Seasonal Influenza Immunization Program
Letter to Elicit Support from Managers (Step 2)

Date

Name
Address

Dear: _______

Re: Support and Involvement of Managers in the Annual Healthcare Personnel Influenza Immunization Campaign

Experience and research has shown that successful immunization programs engage champions at all levels. Support from managers has demonstrated positive outcomes and increased rates.

The best evidence points to the advantages of obtaining the highest possible healthcare personal influenza immunization rate, which is often quoted as 95% to 100%.

Despite abundant evidence about the safety and effectiveness of the influenza vaccine, last year our organization achieved an immunization rate of ___%.

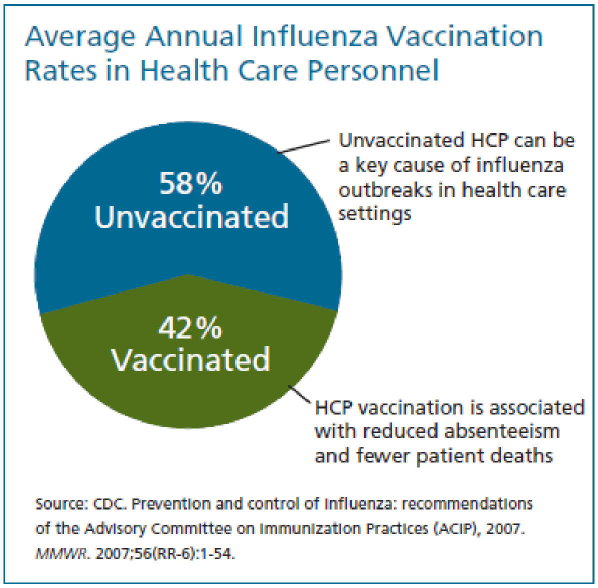

Source: CDC. Prevention and control of influenza: recommendations of the Advisory Committee on Immunization Practices (ACIP), 2007. *MMWR*. 2007;56(RR-6):1-54.

We need your support to assist with the promotion of our annual influenza immunization campaign.

We are requesting your support to assist with the promotion of our annual influenza immunization campaign. Your support is needed to make sure the staff immunization policy is followed; to attend the campaign launch; to lead by example by getting immunized and to encourage healthcare personnel immunization by highlighting the importance of influenza immunization at meetings.

Together we can reach our target and protect our patients/residents.

Respectfully,

Influenza Immunization Program Planning Committee Chair

Credit: Canadian Healthcare Personnel Influenza Immunization Network, 2012

Canadian Healthcare Influenza Immunization Network
Réseau Canadien d'Immunisation contre l'Influenza dans les Soins de Santé
www.chiin.ca

Healthcare Personnel Seasonal Influenza Immunization Program
Policy for Human Resource Management in a Confirmed Influenza Outbreak
(Step 2)

Preamble

In the spirit of “doing no harm” and to meet expectations of a patient-safety oriented healthcare system, all healthcare personnel have a responsibility to take all reasonable action to avoid influenza transmission to those in their care. Healthcare personnel are in a unique position to act as carriers for influenza whether or not symptoms are present. Patients / Residents are vulnerable and at high risk of developing serious influenza-related complications that can be fatal.

Goal

The goal of this policy is to support the organization to reduce influenza transmission to persons at high risk of developing serious and possibly fatal complications related to influenza.

Policy

This policy applies to all persons who carry on activities in this organization including; employees, students, medical residents, physicians, volunteers, pastoral care and contract workers (or collectively “healthcare personnel”. See definition below).

In the event of an influenza outbreak:

In the event of a confirmed outbreak of influenza in the facility, as confirmed by <insert name ___ public health department or _____Infection Control Program, every effort will be made to ensure the health of all in the facility.

When an outbreak has been confirmed:

1. Healthcare personnel must provide proof of immunization on request
2. Unit/ward managers and occupational health are responsible for identifying immunization status of all healthcare personnel
3. Occupational Health / Infection Control will provide advice regarding immunization/antiviral management of unimmunized staff
4. Unimmunized staff must be offered immunization and antivirals

Healthcare personnel (HCP) will be allowed to continue to work in the affected area; subject to the following:

5. If HCP has received a flu shot 2 (two) or more weeks before the outbreak began, and may continue to work as long as he/she remains symptom free.

6. If the HCP received the flu shot less than 2 weeks prior to the outbreak, he/she may take an antiviral medication until the vaccine takes effect 2 weeks after the shot.
7. If the HCP has not had the flu shot, he/she may get one and take antiviral medication for 2 weeks until the vaccine has taken effect.
8. In the rare event that a HCP has an unmanageable medical contraindication to the vaccine (and only in this instance), antiviral prophylaxis as an alternate to immunization may be considered for the duration of the outbreak period, but must be taken every day for the duration of the outbreak.
9. Individuals who choose to take the antiviral medication may return to work 4 hours after the first dose and may continue to work as long as he/she is symptom-free.
10. Healthcare personnel who take the antiviral medication in conjunction with the influenza vaccine during an outbreak will either have their prescription paid for <insert your organization's method for providing antivirals>.

    See Appendix B for a sample decision making flow chart.

    During an outbreak it would be ideal for non-immunized HCPs not taking antivirals to be re-assigned to patient care wards/units without an influenza outbreak. Due to varying human resource structures and staff availability this may not be a workable solution.
11. A non-immunized HCP not taking antiviral prophylaxis must recognize that their inability to work on a ward/unit in an outbreak situation places and unexpected burden on their co-workers as immunized healthcare personnel would be required to cover shifts. In a sense co-workers will be penalized for following the HCP Influenza Immunization Policy.
12. An asymptomatic, non-immunized HCP not taking antiviral prophylaxis may:
    a. Be re-assigned to another work area if this is possible
    b. Chose to go on unpaid leave of absence until 14 days after receiving the vaccine
    c. Decline immunization and go on unpaid leave until the outbreak is declared over
    d. Continue to work on the outbreak unit only and must strictly adhere to the Use of Personal Protective Equipment During and Influenza Outbreak Guideline (See Appendix A)
13. Monitoring healthcare personal adherence to PPE is the responsibility of the unit manager or delegate.
14. Healthcare personnel who are governed by a collective agreement, the terms of that agreement will be honoured.
15. Healthcare personnel who provide the required documentation of a medical contraindication for the vaccine or antiviral will receive priority re-assignment to other work units if work is available. If work is not available, they will be on paid leave until work is available on another ward/unit or until the outbreak is over.
16. Once an outbreak is declared over, HCP taking antivirals will be advised by their manager/designate that they are to stop taking the antiviral.

Definitions

Healthcare personnel

The term healthcare personnel (HCP) is defined broadly as all paid and unpaid persons working in healthcare settings.

Healthcare organization (or setting)

Healthcare personnel work in a variety of settings, including (but not limited to) acute care hospitals, long-term care facilities, skilled nursing facilities, rehabilitation centres, urgent care centres, outpatient clinics, home and community healthcare agencies, and emergency medical services.

Valid medical exemptions to influenza immunization

- Persons with a history of an anaphylactic reaction to a previous dose of the vaccine or to any component of the vaccine
- Persons with a history of Guillain-Barré Syndrome

For more information about this policy please contact <name and contact information>.

Appendix A: Use of Personal Protective Equipment During an Influenza Outbreak

High influenza immunization rates are a cornerstone for the prevention of transmission of influenza in healthcare settings.

The use of personal protective equipment (PPE) is a less than optimal alternative to immunization in conjunction with antivirals during an influenza outbreak.

However, the following guidelines are provided for employees who must work during an influenza outbreak without the protection of influenza immunization and antivirals.

1. Gloves

2. Masks - fluid resistant surgical or procedure masks must be worn at all times during activities related to care of patients.

    a. Fit testing may be required

    b. A mask must be removed when:

        i. it becomes moist

        ii. it is hard to breath in

        iii. it is physically damaged (i.e. the strap is broken)

        iv. it is visibly soiled

    c. Masks, once removed, must not be worn again

3. Eye protection must be worn at all times during activities related to care of patients.

4. All PPE must be removed and discarded or sent to processing, following hand washing guidelines for this activity.

5. When not providing care, and not in patient room, the unprotected employee must continue to wear the mask, and practice accepted hand hygiene practices.

6. When leaving the patient area, the unimmunized employee must remove PPE, as discussed above, wash hands and put on a new mask.

These interventions are not necessary if an individual has been immunized and is presently on an antiviral during the period in which the vaccine is not yet protective.

Appendix B – Decision Making Tree – Immunization, Antivirals, Work Reassignment

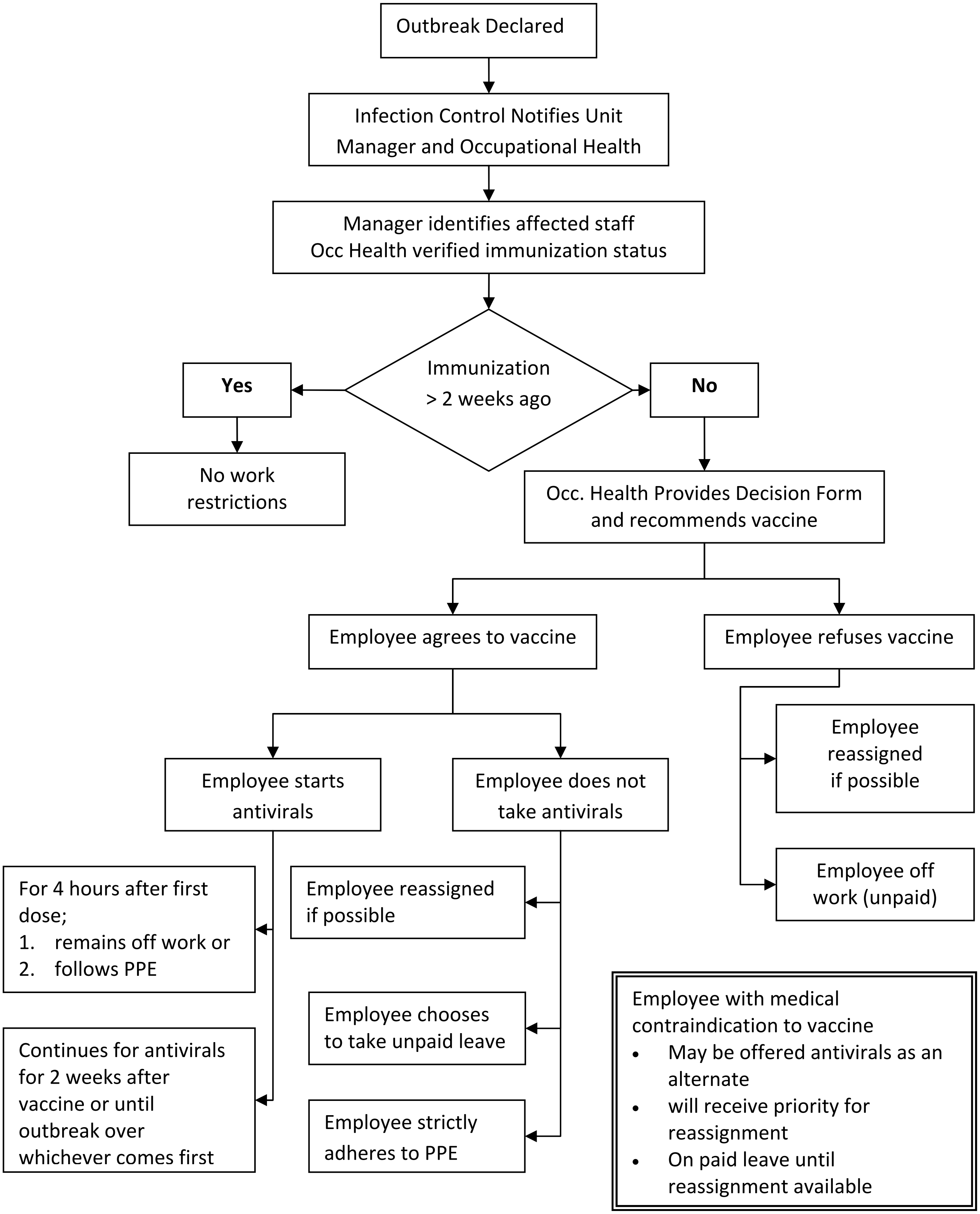

Canadian Healthcare Influenza Immunization Network
Réseau Canadien d'Immunisation contre l'Influenza dans les Soins de Santé
www.chiin.ca

Seasonal Influenza Immunization Decision Form (Step 2)

Employee Name ______________________________ **Department/Program /Unit** ____________________________

Classification (choose one)

☐ Paid Staff (on payroll)
☐ Physician not on payroll
☐ Indirectly paid staff (paid by outside employer)
☐ Medical resident, fellow or student
☐ Volunteer
☐ Other

Patient Contact Category (choose one)

☐ Category I — regular, close, face-to-face, hands on contact
☐ Category II — regular, day-to-day, face-to-face (but not hands-on)
☐ Category III — regularly provide services in patient care areas
☐ Category IV — no patient contact, but performs vital to patient service functions
☐ Category V — no patient contact

Influenza or “flu” is a serious infection of the respiratory tract that causes sickness in millions of people every year. Influenza is spread from person-to-person by coughing and sneezing. Influenza affects all age groups and can lead to severe illness, increased absenteeism from school and work. If you do get influenza, you can be ill and off work for seven days or more. It can also result in other diseases such as pneumonia, hospitalization, and death.

Insert your Organization’s name is committed to providing the best care for our patients and our personnel, which is why we are offering the “flu shot” to all personnel.

Here are some things to keep in mind:

- You cannot get influenza from the vaccine.
- You can be a carrier and spread severe illness to others including your family, the people you work with and our patients, even if you never get sick.
- Immunization provides protection for the whole season against most influenza viruses.
- Side effects to the vaccine are generally mild, and may include a sore arm, mild fever and muscle aches and feeling tired for a day or two.
- Severe allergic reaction is extremely rare.
- The influenza virus usually changes each year which is why there is a different vaccine each year.

………

Please choose one of the following options, sign and return this form to your manager before *insert date*

I have read and understand the information provided to me about influenza and know I can refer to the Employee Influenza Immunization Policy by *insert the method where employees can find your policy*. I have made the following decision:

YES - I choose to be immunized.

☐ I consent to being immunized by Occupational Health and Safety (OHS) officials in my organization during the seasonal influenza campaign and agree to report any unexpected adverse event following immunization to OHS.
☐ I plan to be immunized elsewhere and will bring in or fax to XXX-555-6666, proof of my immunization as soon as I have received the influenza vaccine.

NO – I choose not to be immunized for personal reasons.

☐ I am choosing not to be immunized against influenza at this time. I am aware of the facts detailed above, and understand my organization is committed to immunizing all personnel as part of its commitment to infection prevention and workplace health. I understand that in the event of an influenza outbreak, there may be repercussions (such as work reassignment, wearing personal protective equipment) as outlined in my organization’s influenza immunization policy.

NO – I choose not to be immunized because a physician has confirmed that (please check all that apply):

☐ Yes ☐ No — I am severely allergic to eggs or egg products
☐ Yes ☐ No — I am allergic to neomycin or Thimerosal (preservative)
☐ Yes ☐ No — I have had a previous, confirmed, serious allergic reaction to an influenza vaccine

Signature ____ **Date** ______________________________________

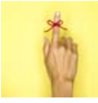

Reminder: In the event of an influenza outbreak, you may be required to show proof of immunization to management by presenting your “proof of immunization” card or other documentation identifying date of immunization. Your immunization status will be released by Occupational Health and Safety Services to *insert the correct information for your organization.*

For Occupational Health Services Use							
Date	Vaccine	Lot#	Manufacturer	Dose	Route	Deltoid	Administered by (nurses signature)
						☐ Left<br>☐ Right	

Credit: Canadian Healthcare Influenza Immunization Network, 2012

Canadian Healthcare Influenza Immunization Network

Réseau Canadien d'Immunisation contre l'Influenza dans les Soins de Santé

www.chiin.ca

Healthcare Personnel Seasonal Influenza Immunization Program
Letter to Elicit Support from Physicians (Step 3)

Date

Name
Address

Dear: _______

Re: Support and Involvement of Medical Directors and Physicians in the Annual Healthcare Personnel Influenza Immunization Campaign

Experience and research has shown that successful immunization programs engage champions at all levels. **There is strong evidence that physician support leads to positive outcomes and increased immunization rates among healthcare personnel.**

The best evidence points to the advantages of obtaining the highest possible healthcare personal influenza immunization rate, which is often quoted as 95% to 100%. Despite abundant evidence about the safety and effectiveness of the influenza vaccine, last year our organization achieved an immunization rate of ___%.

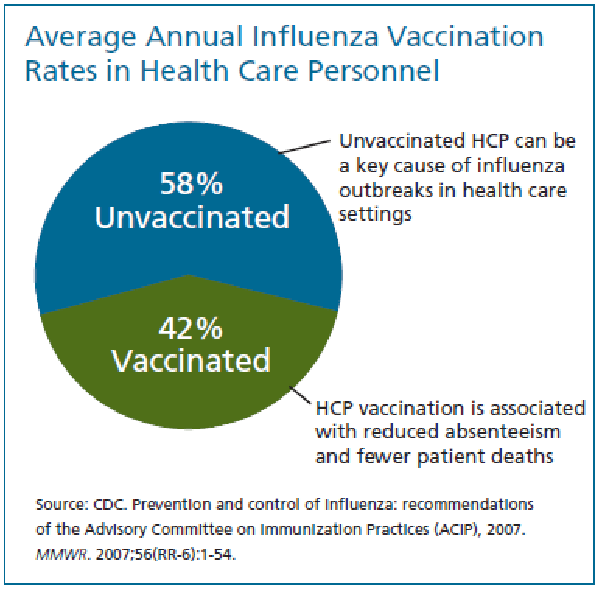

We need your support to assist with the promotion of our annual influenza immunization campaign.

We are requesting your support to assist with the promotion of our annual influenza immunization campaign. You can provide support in a number of ways. Lead by example; attend the campaign launch; get immunized. Encourage peers and colleagues to support the influenza campaign.

Together we can reach our target and protect our patients/residents.

Respectfully,

Influenza Immunization Campaign Planning Committee Chair

Credit: Canadian Healthcare Influenza Immunization Network, 2012

Canadian Healthcare Influenza Immunization Network

Réseau Canadien d'Immunisation contre l'Influenza dans les Soins de Santé

www.chiin.ca

Healthcare Personnel Seasonal Influenza Immunization Program
Letter to Elicit Support from Managers (Step 3)

Date

Name
Address

Dear: _______

Re: Support and Involvement of Managers in the Annual Healthcare Personnel Influenza Immunization Campaign

Experience and research has shown that successful immunization programs engage champions at all levels. Support from managers has demonstrated positive outcomes and increased rates.

The best evidence points to the advantages of obtaining the highest possible healthcare personal influenza immunization rate, which is often quoted as 95% to 100%.

Despite abundant evidence about the safety and effectiveness of the influenza vaccine, last year our organization achieved an immunization rate of ___%.

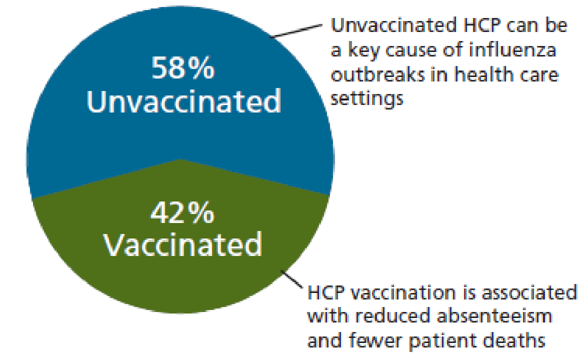

Source: CDC. Prevention and control of influenza: recommendations of the Advisory Committee on Immunization Practices (ACIP), 2007. *MMWR*. 2007;56(RR-6):1-54.

We need your support to assist with the promotion of our annual influenza immunization campaign.

We are requesting your support to assist with the promotion of our annual influenza immunization campaign. Your support is needed to make sure the staff immunization policy is followed; to attend the campaign launch; to lead by example by getting immunized and to encourage healthcare personnel immunization by highlighting the importance of influenza immunization at meetings.

Together we can reach our target and protect our patients/residents.

Respectfully,

Influenza Immunization Campaign Planning Committee Chair

Credit: Canadian Healthcare Influenza Immunization Network, 2012

Healthcare Personnel Seasonal Influenza Immunization Program
Resource Calculation Form (Step 4)

**In-Kind Support**							
**Position/Name**	**Goal**	**Tasks**	**Time commitment**	**What background info do you need first?**	**Who will make the request and how will they do it**	**Follow-up needed by**	**Activity assigned to**
	**(be specific)**	**(itemize each specific task)**	**(be clear how much time you need and when)**		**(in person, via email, using another person to make the request)**	**(insert date)**	**(make sure someone is assigned to follow up)**
CEO/senior VP support	Increase visibility of campaign	Speak to senior managers and VPs	15 minute meeting to persuade / 10 minutes at senior management meeting on 3 Feb 11	Need to speak with infection control manager and make joint presentation / need to research importance of senior management support and impact on campaign	Jean will book a meeting with CEO and IPC manager	January-25-11	Jean will report back to committee
Data analysis support (epidemiology and data support staff)							
Administrative support							
Communications							
Immunizers							
Free promotional items from Public Health / other sponsors							
Budget preparation (i.e. support to track costs)							
Other							

Credit: Canadian Healthcare Influenza Immunization Network, 2012

Healthcare Personnel Seasonal Influenza Immunization Program							
**Resource Calculation Form**							
**Financial Support**							
**Itemized Expenses**	**Goal**	**Amount needed**	**Justify**	**Source**	**Who will present the request, to whom and when?**	**Follow-up needed by**	**Activity assigned to**
	**(be specific)**	**(itemize the specific costs)**	**(clearly outline why you are making a financial request)**	**(who will fund the request, which departments, outside sponsor)**		**(insert date)**	**(make sure someone is assigned to follow up)**
Communications costs (i.e. graphic designer/translator)	Improve communications with French speaking staff	$750	Need to translate decision & consent forms at $.22 / word	Request from admin	Jean will ask the CEO at senior management meeting, 23 Mar 11	Jean will advise committee of results on 3 Apr 11	Jean
Information technology (could be a one–time cost)							
Additional nurses/physicians to give the shots							
Promotional items							
Administrative support							
Other: (specify)							
	**Total Cost**	$750					

Credit: Canadian Healthcare Influenza Immunization Network, 2012

Healthcare Personnel Seasonal Influenza Immunization Program
Resource Calculation Form

In-Kind Support

Position/Name	Goal	Tasks	Time commitment	What background info do you need first?	Who will make the reqeust and how will they do it?	Follow-up needed by	Activity assigned to
	**(be specific)**	**(itemize each specific task)**	**(be clear how much time you need and when)**		**(in person, via email, using another person to make the request)**	**(insert date)**	**(make sure someone is assigned to follow up)**
CEO/senior VP support	Increase visibility of campaign	Speak to senior managers and VPs	15 minute meeting to persuade / 10 minutes at senior management meeting on 3 Feb 11	Need to speak with infection control manager and make joint presentation / need to research importance of senior management support and impact on campaign	Jean will book a meeting with CEO and IPC manager	January 25, 2011	Jean will report back to committee
Data analysis support (epidemiology and data support staff)							
Administrative support							
Communications							
Immunizers							
Free promotional items from Public Health / other sponsors							
Budget preparation (i.e. support to track costs)							

Financial Support

Itemize Expenses	Goal	Amount Needed	Justify	Source	Who will present the request, to whom and when?	Follow-up needed by	Activity Assigned to
	**(be specific)**	**(itemize the specific costs)**	**(clearly outline why you are making a financial request)**	**(who will fund the request, which departments, outside sponsor)**		**(insert date)**	**(make sure someone is assigned to follow up)**
Communications costs (i.e. graphic designer/translator)	Improve communications with French speaking staff	$750	Need to translate decision & consent forms at $.22 / word	Request from admin	Jean will ask the CEO at senior management meeting, 23 Mar 11	Jean will advise committee of results on 3 Apr	Jean
Information technology (could be a one–time cost)							
Additional nurses/physicians to do give the shots							
Promotional items							
Administrative support							
Other: (specify)							
**Total Cost**		$750					

Credit: Canadian Healthcare Influenza Immunization Network, 2012

Healthcare Personnel Seasonal Influenza Immunization Program
Post-Campaign Announcement (Step 5)

The following text can be used in:

- An employee newsletter
- Within an organization wide e-mail
- Enclosed with a pay stub

Influenza Immunization Campaign Report

[We are pleased to announce that [number/percentage of] healthcare personnel were immunized against influenza this year.

We appreciate the commitment of everyone who took the time to be immunized and everyone who helped make the clinics and campaign happen. We are proud of our work this year in keeping everyone healthier.

Congratulations go out to the following departments/units that achieved the highest percentage of their staff immunized (or you can highlight the ones that made the most improvement):

1. [Department], [number/percent of staff vaccinated]
2. [Department], [number/percent of staff vaccinated]
3. [Department], [number/percent of staff vaccinated]

Remember, influenza immunization is only effective for one year.
See you again next year!

Adapted from: Massachusetts Medical Society - ***Employee Flu Immunization Campaign Kit***
Retrieved July 28, 2009 from:
http://www.massmed.org/AM/Template.cfm?Section=Flu&TEMPLATE=/CM/HTMLDisplay.cfm&CONTENTID=11884

Credit: Canadian Healthcare Influenza Immunization Network, 2012

Canadian Healthcare Influenza Immunization Network

Réseau Canadien d'Immunisation contre l'Influenza dans les Soins de Santé

www.chiin.ca

Healthcare Personnel Seasonal Influenza Immunization Program
Post-Campaign Assessment Worksheet (Step 5)

Step 1

Assessment of This Year's Efforts	Yes	No	Ways to Improve Next Year
1. Did you use a multidisciplinary planning team?			
2. Did you include opinion leaders and senior managers?			
3. Have you designated a team member to coordinate the marketing and communications functions?			
4. Which departments or disciplines had the least number of staff members receiving their influenza immunization? (e.g. physicians, nurses, housekeepers, maintenance personnel etc.)?<br>______________________________<br>______________________________<br>______________________________			
5. Were these groups represented on your planning committee?			

Credit: Canadian Healthcare Influenza Immunization Network, 2012

Step 2

Assessment of This Year's Efforts	Yes	No	Ways to Improve Next Year
1. Did you have a HCP immunization policy?			
2. Did you require personnel to report their immunization status?			
3. Did you require personnel to sign a form if they chose not to be vaccinated (decision/consent form)?			
4. Did you use immunization champions or role models?<br><br>• At the senior management level?<br>• At the management level?<br>• At the unit / staff level?			

Credit: Canadian Healthcare Influenza Immunization Network, 2012

Step 3

<table>
<tr><th>Assessment of This Year’s Efforts</th><th>Yes</th><th>No</th><th>Ways to Improve Next Year</th></tr>
<tr><td>1. Did you use methods to improve immunization access?

• Kick-off
• On-site clinic
• Rolling cart clinics
• Other: ______________________</td><td></td><td></td><td></td></tr>
<tr><td>2. Did you use tools for campaign promotion and staff education?

• Flyers/handouts
• Posters
• Email
• Employee newsletters
• Articles from newspapers / publications / on-line sites
• Information attached to pay stubs before campaign / during campaign / post campaign
• In-service training
• Other: ______________________</td><td></td><td></td><td></td></tr>
<tr><td>3. Were incentives or rewards used to engage the undecided?

• Departmental competitions
• Refreshments
• Raffle
• Games
• Other: ______________________</td><td></td><td></td><td></td></tr>
<tr><td>4. Were you able to establish an evaluation framework for your immunization program?</td><td></td><td></td><td></td></tr>
<tr><td>5. Did you complete a SWOT analysis?

• Strengths
• Weaknesses
• Opportunities
• Threats</td><td></td><td></td><td></td></tr>
</table>

Step 4

Assessment of This Year's Efforts	Yes	No	Ways to Improve Next Year
1. Were you able to establish a method to track your immunization progress?			
2. Are you reflecting your numbers using a rate?			
3. Are you tracking all staff?			
4. Is the tracking system flexible?			
5. Were you able to identify and secure the human and financial resources required?			

Step 5

Assessment of This Year's Efforts	Yes	No	Ways to Improve Next Year
1. Were immunization rates shared with staff?			
2. Number of employees immunized this year ___________			
3. Were you able to identify personnel by position / classification / or patient contact category?			
4. Did you plan a post-campaign event to celebrate?			

Credit: Canadian Healthcare Influenza Immunization Network, 2012

Canadian Healthcare Influenza Immunization Network

Réseau Canadien d'Immunisation contre l'Influenza dans les Soins de Santé

www.chiin.ca

Healthcare Personnel Seasonal Influenza Immunization Program
Process Evaluation Template (Step 5)

To conduct a process evaluation, ask your program team members to evaluate how they think they did based on the five steps to planning an effective influenza immunization program.

Please see the Guide for ideas related to selecting measurement indicators.

Step	Selected Measurement Indicator	Met	Unmet
1. Identify and Engage Your Program Team			
2. Outline Your Implementation Plan			
3. Determine Appropriate Components and Relevant Tools			
4. Secure Resources, Implement, and Monitor			
5. Evaluate and Celebrate			

Where an indicator has not been met, provide an explanation as to why indicator was not met and what approach might be used to meet the indicator next year.

Credit: Canadian Healthcare Influenza Immunization Network, 2012
