# Supplementary material for: A New Approach to Improving Healthcare Personnel Influenza Immunization Programs: A Randomized Controlled Trial
Source: PLoS One. 2015 Mar 17;10(3):e0118368. doi: 10.1371/journal.pone.0118368 (PMC4363667; doi:10.1371/journal.pone.0118368)
Supplement: S1 Protocol — (DOC) [file pone.0118368.s007.doc]

**A Cluster Randomized Controlled Trial to Evaluate the Effectiveness of the “Successful Influenza Immunization Campaigns for Healthcare Personnel: A Guide for Campaign Planners”**

**A Component of the “Optimizing Healthcare Workers Interpandemic Vaccine Uptake in Acute and Long-Term Care” CIHR Project**

**Principal Investigators: Dr. Larry W. Chambers**

**Dr. Anne McCarthy**

**Co-Investigators: Dr. Shelly McNeil**

**Dr. Donna Pierrynowski MacDougall**

**Dr. Virginia Roth**

**Dr. Kathryn Suh**

**Dr. Kumanan Wilson**

**Ms. Donna Baker**

**Ms. Lois Crowe**

**Ms. Sarah DeCoutere**

**Ms. Heather Hall**

**Ms. Po-Po Lam**

**Ms. Elina Haynes**

**Ms. Caroline George**

**Date: August 1, 2010**

**Sponsor: Canadian Institutes of Health Research**

**1.0 Introduction**

1.1 Background

The Canadian Institutes of Health Research (CIHR) has awarded our project team three years of support for the research project entitled “Optimizing Healthcare Worker Interpandemic Vaccine Uptake in Acute and Long-Term Care”. The goals of this project are to 1) evaluate the use of the Ottawa Influenza Decision Aid (OIDA) as an adjunct to occupational influenza immunization campaigns in acute and long term healthcare organizations; 2) to develop and evaluate the use of a Guide for influenza immunization campaigns including methods to integrate the OIDA into a campaign; and 3) to establish a national knowledge exchange network to improve best practices in influenza immunization among healthcare personnel. We describe here the components of the project involving the implementation and evaluation of the Guide “Successful Influenza Immunization Campaigns in Healthcare Organizations: A Guide for Campaign Planners” to be used by healthcare organizations to plan their seasonal influenza immunization campaign for healthcare personnel.

Keeping nurses, physicians and allied health professionals working during seasonal influenza epidemics is essential. Influenza immunization has been shown to mitigate the adverse health outcomes associated with influenza in the community. When provided to healthcare personnel, influenza vaccine reduces the frequency and severity of nosocomial influenza outbreaks and reduces influenza-associated morbidity and mortality among patients by reducing the transmission of influenza from healthcare personnel to their vulnerable patients (1).

Influenza is one of the leading causes of acute respiratory infection and results in significant increases in average life-years lost, hospital lengths of stay and outpatient clinic visits (2-4), representing an enormous economic burden for many countries (5). Healthcare personnel are at high risk of exposure to influenza as they are exposed in both the community and in the workplace (6). Influenza immunization for healthcare personnel has been shown to reduce patient mortality in long-term care facilities as well as employee absenteeism and financial costs in acute care settings (7-12). The Canadian National Advisory Committee on Immunization (NACI) recommends that influenza immunization campaigns strive to immunize at least 90% of eligible recipients (13). In fact, NACI regards influenza immunization of healthcare personnel with direct patient care responsibilities as an essential component of the standard of care for the protection of patients and the refusal of healthcare personnel without contraindications to receive influenza vaccine as a failure in their duty of care to patients. Despite national recommendations and proven effectiveness, influenza immunization coverage rates among healthcare personnel remain below targeted levels (13).

1.2 Poor Uptake of Influenza Vaccine by Healthcare Personnel

Despite abundant evidence of the safety and efficacy of the influenza vaccine, immunization rates among healthcare personnel in hospital and long-term settings remain well below the public health target of 90%. Current initiatives targeted at increasing healthcare personnel immunization rates are having limited success with healthcare personnel immunization rates as low as 2%, but averaging 40% - 60% in most healthcare facilities being reported (1,2,12).

1.3 Development of the Guide: “Successful Influenza Immunization Campaigns for Healthcare Personnel - A Guide for Campaign Planners”

Healthcare organizations have a wide variety of approaches to the immunization of their workers. Each influenza season, healthcare planners devote time and effort to encourage staff immunization through a seasonal influenza immunization campaign. While the majority of healthcare organizations are committed to vaccinating their personnel, they, however, work in isolation, with few resources and limited evidence to inform the development and running of a successful immunization campaign. Moreover, there are almost no additional resources available and often few opportunities to share ideas, ask questions, or boast successes with other organizations (14).

During the first year of the project team’s grant (2008-2009), we conducted a series of consultation meetings in Ontario and Nova Scotia with five long-term care homes and three acute care hospitals. We spoke to the influenza campaign teams typically comprising of occupational health and infection control leaders as well as other managers. The results of these consultation meetings led to the first draft of the Guide: “Successful Influenza Immunization Campaigns for Healthcare Personnel: A Guide for Campaign Planners” (see Appendix 1).

The goal of this study is to validate the Guide and additional web-based “grab and go” tools (the Toolkit).

The Guide is structured to facilitate the use of the evidence-based research on strategies to increase immunization rates by healthcare planners. It also introduces ways to incorporate the Ottawa Influenza Decision Aid (OIDA, another tool in produced by the project team) into their campaign. The Guide is based on the following sources of information: 1) Systematic review of literature on interventions to increase healthcare personnel immunization rates (15); 2) ideas from consultation meetings with campaign organizers, 3) experiences of organizations implementing the OIDA in 2008-2009, 4) results from the OIDA pilot studies, 5) input from Occupational Health and Infection Control practitioners working closely with the project team and 6) additional research by team members on specific topics within the Guide.

In 2009, this Guide was pilot-tested and updated based on the feedback that was received. During the 2009-2010 influenza campaign, despite the impact of H1N1, sixteen healthcare organizations agreed to use and evaluate the Guide. Those who used the Guide during the pilot test reported that the Guide had a positive impact on their program, with 100% indicating they would recommend it to other organizations and 90% stating they made changes to their organization’s influenza immunization campaign for their healthcare personnel based on the information contained in the Guide.

Following completion of the study, we will work with partner organizations to make these tools available to all Canadian healthcare organizations.

Comments received from users of Version One of the Guide:

“Provides a structured and planned approach to ensure everything is covered”

“Helped us to approach campaign in a planned way”

“Provided pre-planning ideas that resulted in better communication and dialogue from employees”

“I felt better prepared to research new resources and build on old ones”

| **What they learned …** |
| --- |
| - Importance of providing education to employees (i.e. risks, benefits of flu shot) - Necessity of identifying resources required - Importance of good communication plan - Importance of pre-planning and post-campaign evaluation and feedback - Incentives - Calculation of statistics - Getting involvement from others - Use of informed declination - Increase role of communications and human resources at all stages of campaign - More than 1-2 components (methods) used to increase participation - The need to review policies and procedures - Identification of barriers and how to overcome them - Identification of what you can improve as a facility and an organization - Validated and clarified what we had in place |
| **What they liked …** |
| - Checklist at front is excellent - Self-explanatory – clean and concise - Use of expanded team - SWOT (Strengths, Weakness, Opportunities, Threats) - A lot of new things, planning guide very helpful - Declination form - OIDA - Influenza season – approach, making it sensible and evaluating what I could do to improve - Very helpful (especially the section on identifying your team) |

During the 2010–2011 and the 2011-2012 seasonal influenza immunization campaigns, we will recruit a minimum of 46 healthcare organizations to either conduct their campaigns as usual or to use the Guide and the Toolkit. At the end of the campaign season we will evaluate the impact of the Guide and web-based tool and assess whether there were changes in immunization rates within each organization and/or if there were significant changes in the management of the campaigns across organizations.

Successful Influenza Immunization Campaigns in Healthcare Organizations: A Guide for Campaign Planners (the Guide) is designed for:

- Healthcare organizations committed to improving immunization rates
- The person or team responsible for implementing the campaign within their organization (or component of a healthcare organization)

The evidence-based Guide includes:

- Checklists
- Simple, easy-to-use framework
- Electronic templates (in the Toolkit) that can be customized
- Interactive web-based tools

1.4 Research Question

Among healthcare organizations who conduct Seasonal Influenza Immunization Campaigns, does use of the Guide impact the effectiveness of these campaigns as measured by change of healthcare personnel immunization rates and in an organization’s ability to plan, implement, monitor, and evaluate their campaign?

- 1. Research Objectives

1. To assess any changes in healthcare personnel immunization rates between the Control Group (usual campaign) and the Intervention Group (campaigns using the Guide)
2. To assess if, by tracking immunization rates by direct patient category, healthcare organizations are better able identify lower or higher immunization rates among the healthcare personnel who have the most direct patient contact (16)
3. To assess the effectiveness of the Guide. We will do this by assessing the Intervention Groups’ ability to make the changes in campaign planning, implementation, monitoring, and evaluation using the recommendations in the Guide
4. We will assess the delivery methods of the Guide (paper, web-based) and assess the Intervention Group’s satisfaction with ease of use, practicality of application, and need for outsider intervention
5. **Methods**
   1. Trial Design

This will be a multi-centre, cluster, randomized controlled trial.

2.2 Rationale for a Cluster Randomized Control Trial

Our intention in choosing a cluster randomized control trial design is to evaluate the impact of a tool designed for use at an organizational level, rather than an individual level. In order to understand its impact we will assess changes within each organization according to the research objectives detailed herein.

1. Participants & Study Setting

At least 46 healthcare organizations from across Canada will be selected to participate in the study.

The Guide is designed to be used across different types of healthcare organizations. Potential healthcare organizations will be self-identified, recommended by a project team member, and/or selected from the Canadian Healthcare Association’s Guide to Healthcare Facilities. Selected sites must meet the following eligibility criteria:

- - Canadian healthcare organizations who conduct seasonal influenza immunization campaigns
  - Have a systematic approach to collecting healthcare personnel immunization rates and be able to provide accurate and timely reports of immunization rates for categories of healthcare personnel
  - Agree to be randomized to receive the Guide or receive no intervention
  - If randomized to receive the Guide, the organization, including the campaign team and senior management, agrees to commit to adhere to the steps in the Guide to plan, implement, monitor and evaluate their campaign

Influenza immunization campaigns in healthcare organizations in Canada normally begin in October and end in December, with ongoing influenza immunization continuing outside of the reporting parameters until April.

1. **Interventions**

All organizations who agree to participate in the study will agree to provide their immunization rates for three years:

2008-2009 – Baseline Year

2010-2011 – Year One

2011-2012 – Year Two

We did not choose 2009-2010 as the baseline year because of the impact of pandemic H1N1.

- 1. Intervention Group

For the intervention group, there will be a Guide facilitator provided by the project team, normally the Project Manager or their delegate. The role of the Guide facilitator will be to provide basic information about the Guide and to facilitate the use of the web-based tools.

This Intervention Group will be invited to participate in either a half-day workshop or Webinar in September 2010 and September 2011. Ideally, this will be done in partnership with the Canadian Coalition on Immunization Awareness and Protection (CCIAP), public health and regional health authorities. The purpose of this intervention is to provide a basic introduction to the Guide and introduce the Guide Facilitator. The Intervention Group will be given access to the website and access to the Guide.

In March 2011 and again in March 2012, they will be asked to report their influenza immunization rates and complete an evaluation of the Guide.

In October, January and March of each campaign year, there will be a Webinar or Teleconference with the Intervention Group to answer questions, share challenges, and establish a community of practice. The Group, or subsets of the Intervention Group, may choose to meet more often and will be invited to participate in a web-based chat forum moderated by the Guide facilitator or their delegate.

- 1. Control Group

For the control group, the team will register the organization and will collect their immunization rates, baseline organizational data, and campaign activities for 2008-2009. We will contact them in March 2011 and March 2012 to collect immunization rate data and report on campaign activities. We will ask them open-ended questions to determine the content and process used in their more recent campaigns based on the focus group results from our work with the 16 organizations in 2009-2010.

They will be given access to the public part of the CHIIN website, and will be encouraged to calculate their immunization rates using the methodology in Appendix 3 of the Guide (16). This information will be publically available by September 2010.

The Control Group will be given access to the Guide at the end of the two-year period. In terms of contamination, Control Group participants may be disappointed and seek their own way of increasing immunization rates among healthcare personnel. The final unstructured interviews should capture this information.

- 1. Questionnaires

**Control Group – September 2010**

- Registration Form
- Immunization rate data for 2008-2009

**Control Group – March 2011 and March 2012**

- Immunization rate data for each year
- Answer open-ended question re: campaign activities

**Intervention Group – September 2010**

The following questionnaires will be given before campaign begins:

- Registration Form
- Campaign Details Questionnaire
- Immunization rate data for 2008-2009

**Intervention Group – March 2011 and March 2012**

- Immunization rate data for each year
- Campaign Details Questionnaire
- Assessment of the Guide
- Assessment of the revised OIDA
- Assessment of the Toolkit

All questionnaires will be given as self-administered questionnaires and will be available online or via email. Each questionnaire will include check-box questions (which will be analyzed using descriptive analysis) and open-ended questions. The results from the open-ended questions and information gathered from the workshops, webinars, and teleconferences, plus interactions with the research team throughout the campaign season, will be summarized and synthesized in a post-campaign report each year.

1. **Study Outcomes**
   1. Primary Outcome

In the Intervention Group, a 10% improvement in healthcare personnel immunization rates from the base year (2008-2009) as compared to Year One (2010-2011) and Year Two (2011-2012)

This will be assessed by conducting a time-series analysis on how each organization does against themselves and between the Intervention and Control Group. In our systematic review of the effectiveness of influenza campaigns in healthcare organizations published in the Canadian Medical Association Journal (15) (see Appendix 2), methods for calculating change scores were used that enable comparisons across different types of studies randomized trials with only “after” assessments in the intervention and control groups as well as comparisons of studies that had change scores based on before and after the campaigns in control and intervention groups.

Also, we will assess the immunization rates against the benchmark of 90% set by the National Advisory Committee on Immunization (17).

- 1. Secondary Outcome

In both the Intervention and Control Group, we will assess improvement in the reporting of immunization rates, based on the methodology outlined in Appendix 3 of the Guide (16).

- 1. Tertiary Outcomes

Improvement in how each organization plans, implements, monitors, and evaluates their campaign will be observed. We will track this improvement comparing their campaign activities each year against the benchmarks established by the Checklist in the Guide.

Overall satisfaction with the Guide and with the delivery methods of the Guide, including preferences for web-based or paper tools, ease of use, practicality of application, and the need for outsider intervention, will be assessed.

1. **Sample Size**

The sample size is based on both the number of healthcare organizations and the number of healthcare personnel in each organization. We aim to accumulate data on a minimum of 150 healthcare personnel per cluster over 2 years. Thus, if an organization is in the trial for two years they will have to contribute at least 300 healthcare personnel to the trial. Note that we define a cluster as a healthcare organization. Sample size was calculated based on the equations of Kelsey et al. for Randomized Clinical Trial Studies (18) and multiplied by the design effect to get the number of subjects required for the cluster design. The level of difference between intervention organizations and the control organizations set in determining the sample size was 20%. This requires randomization and evaluation of immunization campaigns in 46 healthcare organizations (clusters) with 6000 healthcare personnel. Twenty-three will be randomized to the intervention group. With an intra-cluster correlation coefficient set at 0.05 and an average cluster size of 150 eligible healthcare personnel per healthcare organization, we will need a total of 46 clusters for 80% power and 5% significance.

- 1. Statistical Methods

As outlined in the sample size calculation for the study, the statistical analysis will be based on analyses using cluster analytic techniques.

- 1. Interim Analysis and Stopping Rules

We will conduct an interim analysis after completion of Year One. There are no stopping rules.

1. **Randomization**
   1. Randomization Sequence Generation

After organizations have declared their willingness to participate in the study, they will be randomized to be in the Intervention Group or Control Group. Due to the relatively small number of healthcare organizations in the trial, minimization will be used to allocate participants to intervention and control to ensure maximum balance. After all participants have been recruited, organizations will be individually allocated to an intervention and/or control group by the Ottawa coordinating centre using computer-generated random number tables, embedded in a computer program for minimization. The variables to be used in the minimization process will be trial centre (Ontario and Manitoba) and healthcare organization size (small, medium, large).

Organizations assigned to the control group will be informed that they will have access to the Guide at the completion of the study.

- 1. Randomization Type

Randomization will be performed using complete simple randomization.

1. **Statistical Methods**

The primary analysis will consist of comparing the Intervention Group with the Control Group. Characteristics of healthcare organizations will be compared descriptively at baseline. In order to adjust for the cluster design, we will calculate mean immunization rates and mean proportions of other characteristics for each healthcare organization to produce summary statistics.

8.1 Primary Outcome

For Year One and Year Two, we will calculate the mean difference in immunization rates between the intervention year and baseline for both comparison groups. Assuming that the mean values are normally distributed within each group, we will use t-test to compare immunization rate difference means of the control group to the intervention group. We will consider *P* values < 0.05 to be significant.

Linear regression models will be used for continuous outcome variables (improvement in healthcare personnel immunization rates from the base year) and logistic models for binary outcome variables (attainment of the benchmark of 90% immunization rate set by the National Advisory Committee on Immunization). The assumptions for using each regression model will be checked and analyses adjusted accordingly. All analyses will be undertaken, adjusted for healthcare organization size, and for baseline variables which differ to a statistically significant extent between groups. Differential effects of the intervention by characteristics of healthcare organization and baseline rates will be assessed for the primary outcome measures by adding terms for the interaction between these characteristics and baseline rates and Intervention Group to the regression models.

Multilevel models will be applied to take account of clustering at the healthcare organization level and the Guide effects which will apply to differing extents in the intervention group (due to the effectiveness of campaigns in each participating healthcare organization). While the primary outcome will be attainment of the recommended benchmark of 90% set by the National Advisory Committee on Immunization, we will investigate the profile of immunization rate attainment at Baseline (2008-2009), Year One (2010-2011) and Year Two (2011-2012). This will be a repeated measures analysis. That is, evidence for differences in effect of the intervention at the different time points will be assessed.

8.2 Secondary Outcome

Descriptive analysis of the campaign questionnaire results will be conducted. Chi-square test will be used to analyze differences between comparison groups for categorical variables and t-test will be used for nominal variables.

Missing outcome data will be assumed to be "missing at random", conditional on key predictors of "missingness" (in particular, Baseline values, Intervention Group, and measures of compliance post-randomisation). Multiple imputations of outcome variables will be carried out using these predictors of "missingness".

1. **Feasibility Study Management**
   1. Day-to-day Management

The Coordinating Centres are located across the two sites, Élisabeth Bruyère Research Institute and the Ottawa Health Research Institute, in Ottawa. Personnel will include the co-principal investigators, project manager, research assistants, statistician, data analysts, and data entry staff. The Coordinating Centres are responsible for the day-to-day management of the trial.

The Guide Facilitator will be the project manager or their delegate, with an effort made to have a provincial facilitator available to coordinate the study in each participating province. The provincial facilitators liaise with each organization, collecting all data and ensuring the organization has access to facilitators as needed.

# Data Management

Data collected from the web-based questionnaires will only be accessible to the project team using a secured password. Discussion notes from the workshops, webinars or teleconferences will not include any personal identifiers and will be kept by the project manager for the duration for the study.

1. **Ethical Issues**

- 1. Research Ethics Board Approvals

Ethics approval of this study will be obtained within the context of the CIHR by the Coordinating Centre sites by The Ottawa Hospital Research Ethics Board and the Bruyère Continuing Care Research Ethics Board. Ethics approval will be obtained from each participating organization only if required by them.

The Research Ethics Boards will be notified of any changes are made to the study protocol, and no activity will take place before ethical approvals are obtained.

- 1. Informed consent

No individual informed consent will be required. This study is a feasibility study of new tools for use by healthcare organizations within their established influenza immunization campaigns. No personal individual information will be collected.

- 1. Confidentiality

1. Some sites in the Intervention Group may choose to collect the OIDA and send to the Coordinating Centre for reference. No personal information is collected on the OIDA and its completion is completely anonymous.
2. All study data will be anonymized. Electronic data will only be accessible by appropriate study personnel and password protected. Digital transcripts will be sent for transcription with an authorized transcriptionist, with all identifying material removed. All paper data is held behind locked doors in locked cabinets and accessible to study personnel only.
3. Any workshop, webinar and teleconference transcripts may be recorded digitally and meeting notes will be taken. After all identifying information has been removed, the transcripts may be used for qualitative analysis, including coding for themes using nVivo software. The digital records will be kept for three years. The paper records will be kept with the rest of the study documentation for 15 years after termination of the study.
4. The names of the organizations that may audit study records include the Ottawa Hospital Research Institute, the Élisabeth Bruyère Research Institute, the Bruyère Continuing Care Research Ethics Board, The Ottawa Hospital Research Ethics Board, and the Canadian Institutes of Health Research.
5. The study records will be kept for 15 years after termination of the study. Paper documents will be shredded and electronic files deleted.

#

1. **Publication of Results**

The results of this study will be published in peer-reviewed healthcare journals and will be presented at scientific conferences.

**References**

1. Harper SA, Fukuda K, Uyeki TM, Cox NJ, Bridges CB, Centers for Disease Control and Prevention (CDC) Advisory Committee on Immunization Practices (ACIP). Prevention and control of influenza: recommendations of the Advisory Committee on Immunization Practices (ACIP). MMWR Recomm Rep. 2004 May 28;53(RR-6):1-40.
2. Cunney RJ, Bialachowski A, Thornley D, Smaill FM, Pennie RA. An outbreak of influenza A in a neonatal intensive care unit. Infect Control Hosp Epidemiol. 2000 Jul;21(7):449-454.
3. Maltezou HC, Drancourt M. Nosocomial influenza in children. J Hosp Infect. 2003 Oct;55(2):83-91.
4. Sartor C, Zandotti C, Romain F, Jacomo V, Simon S, Atlan-Gepner C, et al. Disruption of services in an internal medicine unit due to a nosocomial influenza outbreak. Infect Control Hosp Epidemiol. 2002 Oct;23(10):615-619.
5. Molinari NA, Ortega-Sanchez IR, Messonnier ML, Thompson WW, Wortley PM, Weintraub E, et al. The impact of seasonal influenza in the US: measuring disease burden and costs. Vaccine. 2007 Jun 28;25(27):5086-5096.
6. Hofmann F, Ferracin C, Marsh G, Dumas R. Influenza immunization of healthcare workers: a literature review of attitudes and beliefs. Infection. 2006 Jun;34(3):142-147.
7. Carman WF, Elder AG, Wallace LA, McAulay K, Walker A, Murray GD, et al. Effects of influenza immunization of health-care workers on mortality of elderly people in long-term care: a randomised controlled trial. Lancet. 2000 Jan 8;355(9198):93-97.
8. Nichol KL, Lind A, Margolis KL, Murdoch M, McFadden R, Hauge M, et al. The effectiveness of immunization against influenza in healthy, working adults. N Engl J Med. 1995 Oct 5;333(14):889-893.
9. Lester RT, McGeer A, Tomlinson G, Detsky AS. Use of, effectiveness of, and attitudes regarding influenza vaccine among house staff. Infect Control Hosp Epidemiol. 2003 Nov;24(11):839-844.
10. Weinstock DM, Eagan J, Malak SA, Rogers M, Wallace H, Kiehn TE, et al. Control of influenza A on a bone marrow transplant unit. Infect Control Hosp Epidemiol. 2000 Nov;21(11):730-732.
11. Hayward AC, Harling R, Wetten S, Johnson AM, Munro S, Smedley J, et al. Effectiveness of an influenza vaccine programme for care home staff to prevent death, morbidity, and health service use among residents: cluster randomised controlled trial. BMJ. 2006 Dec 16;333(7581):1241.
12. Potter J, Stott DJ, Roberts MA, Elder AG, O'Donnell B, Knight PV, et al. Influenza immunization of health care workers in long-term-care hospitals reduces the mortality of elderly patients. J Infect Dis. 1997 Jan;175(1):1-6.
13. National Advisory Committee on Immunization. Canadian Immunization Guide 7^th^ Edition. Public Health Agency of Canada; 2006.
14. Song JY, Park CW, Jeong HW, Cheong HJ, Kim WJ, Kim SR. Effect of a hospital campaign for influenza immunization of healthcare workers. Infect Control Hosp Epidemiol. 2006 Jun;27(6):612-617.
15. Lam P, Chambers LW, Pierrynowski MacDougall DM, McCarthy AE. Seasonal immunization campaigns for healthcare personnel: systematic review. CMAJ [Internet]. 2010 Jul 19 [cited 2010 Jul 26]. Available from: <http://www.cmaj.ca/cgi/content/abstract/cmaj.091304v1>.
16. Chambers LW et al. Successful Influenza Immunization Campaigns for Healthcare Personnel [guide]. Ottawa: Canadian Healthcare Influenza Immunization Network; 2010.
17. National Advisory Committee on Immunization. Statement on Influenza Vaccination for the 2008-2009 Season. Public Health Agency of Canada; 2008 July.
18. Kelsey JL et al. Methods in Observational Epidemiology. 2^nd^ Ed. New York: Oxford University Press; 1996.
